# Inhibitory Effects of Lipopeptides and Glycolipids on *C. albicans–Staphylococcus* spp. Dual-Species Biofilms

**DOI:** 10.3389/fmicb.2020.545654

**Published:** 2021-01-13

**Authors:** Chiara Ceresa, Maurizio Rinaldi, Francesco Tessarolo, Devid Maniglio, Emanuele Fedeli, Erica Tambone, Patrizio Caciagli, Ibrahim M. Banat, Mayri Alessandra Diaz De Rienzo, Letizia Fracchia

**Affiliations:** ^1^Department of Pharmaceutical Sciences, Università del Piemonte Orientale “A. Avogadro”, Novara, Italy; ^2^BIOtech Center for Biomedical Technologies, Department of Industrial Engineering, Università di Trento, Trento, Italy; ^3^Healthcare Research and Innovation Program (IRCS-FBK-PAT), Bruno Kessler Foundation, Trento, Italy; ^4^Section of Electron Microscopy, Department of Medicine Laboratory, Azienda Provinciale per i Servizi Sanitari di Trento, Trento, Italy; ^5^School of Biomedical Sciences, Faculty of Life and Health Sciences, Ulster University, Coleraine, United Kingdom; ^6^School of Pharmacy and Biomolecular Sciences, Faculty of Science, Liverpool John Moores University, Liverpool, United Kingdom

**Keywords:** biosurfactants, multi-species biofilm, anti-adhesion, anti-biofilm, biomaterials, medical devices

## Abstract

Microbial biofilms strongly resist host immune responses and antimicrobial treatments and are frequently responsible for chronic infections in peri-implant tissues. Biosurfactants (BSs) have recently gained prominence as a new generation of anti-adhesive and antimicrobial agents with great biocompatibility and were recently suggested for coating implantable materials in order to improve their anti-biofilm properties. In this study, the anti-biofilm activity of lipopeptide AC7BS, rhamnolipid R89BS, and sophorolipid SL18 was evaluated against clinically relevant fungal/bacterial dual-species biofilms (*Candida albicans*, *Staphylococcus aureus*, *Staphylococcus epidermidis*) through quantitative and qualitative *in vitro* tests. *C. albicans*–*S. aureus* and *C. albicans*–*S. epidermidis* cultures were able to produce a dense biofilm on the surface of the polystyrene plates and on medical-grade silicone discs. All tested BSs demonstrated an effective inhibitory activity against dual-species biofilms formation in terms of total biomass, cell metabolic activity, microstructural architecture, and cell viability, up to 72 h on both these surfaces. In co-incubation conditions, in which BSs were tested in soluble form, rhamnolipid R89BS (0.05 mg/ml) was the most effective among the tested BSs against the formation of both dual-species biofilms, reducing on average 94 and 95% of biofilm biomass and metabolic activity at 72 h of incubation, respectively. Similarly, rhamnolipid R89BS silicone surface coating proved to be the most effective in inhibiting the formation of both dual-species biofilms, with average reductions of 93 and 90%, respectively. Scanning electron microscopy observations showed areas of treated surfaces that were free of microbial cells or in which thinner and less structured biofilms were present, compared to controls. The obtained results endorse the idea that coating of implant surfaces with BSs may be a promising strategy for the prevention of *C. albicans*–*Staphylococcus* spp. colonization on medical devices, and can potentially contribute to the reduction of the high economic efforts undertaken by healthcare systems for the treatment of these complex fungal–bacterial infections.

## Introduction

Biofilms are complex biological structures, composed of sessile multicellular communities encapsulated in a hydrated matrix of polysaccharides and proteins, in which microorganisms become more resistant to drug therapy and host immune response (Chen and Wen, 2011; Pompilio and Di Bonaventura, 2018). Microbial cells forming biofilms also communicate through the quorum sensing (QS) system, which is responsible of regulating genes expression, production of proteases and other signals that enable high-density bacterial cluster to flourish (Buch et al., 2019; Irorere et al., 2019).

Biofilms give rise to chronic infections both in tissues (e.g., lung infection in cystic fibrosis, chronic wound infections) and on the surface of implantable medical devices (e.g., orthopedic prostheses, endotracheal tubes, intravenous and urinary catheters, heart valves), which are characterized by the development of persistent and progressive diseases mainly due to the inflammatory response surrounding these biofilms (Clinton and Carter, 2015; Percival et al., 2015; Teo et al., 2016; Omar et al., 2017; Song et al., 2018). This makes many biofilm infections difficult to diagnose or to adequately treat.

Although most tissue and medical device-associated infections are caused by a single pathogen, an increasing number of polymicrobial infections have been reported in the clinical practice (Mihai et al., 2015; Rodrigues et al., 2019). The involved microorganisms are believed to coexist and realize synergistic interactions within the biofilm environment resulting in enhanced pathogenicity, virulence, and resistance to antimicrobials, thus leading to more aggressive forms of infections (Marculescu and Cantey, 2008; Burmølle et al., 2014). An emerging finding in polymicrobial biofilm research is the presence of both eukaryotic and prokaryotic pathogens (Brogden et al., 2005). The coexistence of *Candida albicans* and *Staphylococcus* species, in particular, has been frequently associated with extremely complicated infections and high mortality rates (Tsui et al., 2016). Biofilm-associated diseases related to *C. albicans* and *Staphylococcus* species, including wound infections, periodontitis, denture stomatitis, and medical devices related infections involving catheters and orthopedic implants have all been described before (Adam et al., 2002; Gupta et al., 2005; Valenza et al., 2008; Cuesta et al., 2010; Harriott and Noverr, 2011). These polymicrobial infections are difficult to diagnose and are mostly untreatable with the conventional antibiotic treatment strategies and commonly requires complex multi-drug therapy and in the vast majority of cases, the removal of infected medical devices (Harriott and Noverr, 2009; Pammi et al., 2013; Carolus et al., 2019).

This worldwide public health problem requires the development of innovative approaches able to efficiently tackle infections associated with these bacteria, fungi, and their biofilms. For this reason, several surface-coating strategies have been proposed to safeguard medical devices from microbial adhesion and colonization (John et al., 2007; Zilberman and Elsner, 2008; Ramasamy and Lee, 2016; Francolini et al., 2017; Khatoon et al., 2018; Wang and Tang, 2018; Ghensi et al., 2019). Unfortunately, surface-treated biomaterials showed, in some cases, limited efficacy over time as well as an inherent risk of cytotoxicity toward cell tissues (Francolini and Donelli, 2010). Therefore, the application of natural molecules for the creation of new safe and effective biocompatible antibacterial and/or anti-adhesive biomaterial coatings or pharmaceutical products to prevent and treat both single-species and polymicrobial biofilm infections are urgently required (Vasilev et al., 2011).

Focusing on this goal, biosurfactants (BSs) have been suggested as a new group of antimicrobial/anti-biofilm biocompatible compounds useful in a wide range of pharmaceutical and biomedical applications (Diaz De Rienzo et al., 2014; Elshikh et al., 2017; Lydon et al., 2017; Juma et al., 2020). BSs are amphiphilic molecules, produced by a wide group of microorganisms, which partition at and alter the physical–chemical conditions of the interfaces and are characterized by interesting biological activities like antimicrobial, anti-adhesive, and anti-biofilm properties (Banat et al., 2014; Simms et al., 2020). BSs ability to destabilize the integrity and permeability of cell membranes and to modify surface properties of biomaterials, affecting microbial vitality and adhesion, limiting biofilm formation, or reducing the structural integrity of existing biofilms have been reported (Fracchia et al., 2015, 2019; Satpute et al., 2016).

Toward this goal, during the past few years, we have demonstrated the ability of lipopeptides, rhamnolipids, and sophorolipids BSs, alone or in combination with antimicrobials and quorum-sensing molecules to inhibit microbial adhesion and biofilm formation of mono-species biofilms of fungal or bacterial pathogens on biomedical materials such as silicone (Ceresa et al., 2016, 2017, 2018, 2019b, 2020). The activity of BSs against biofilm formation on model surfaces such as polystyrene, glass, silicone, and polydimethylsiloxane has also been described in other works (Sharma and Saharan, 2016; Aleksic et al., 2017; Janek et al., 2018; Satpute et al., 2018). Conversely, to our knowledge, no studies have been conducted yet concerning the use of BSs against these clinically relevant fungal–bacterial polymicrobial biofilms.

In this perspective, the present study aimed at testing the efficacy of three different BSs (lipopeptide AC7BS, rhamnolipid R89BS, and sophorolipid SL18) as anti-adhesive and anti-biofilm agents against the formation of clinically relevant multi-species biofilms composed by fungal and bacterial species (*C. albicans*–*Staphylococcus aureus*, *C. albicans*–*Staphylococcus epidermidis*) employing a multidisciplinary and multifaceted approach.

## Materials and Methods

### Study Design

The study was organized in seven experimental phases: (1) definition of the dual-species biofilm model with quantification of biomass production and metabolic activity on polystyrene and silicone elastomer and comparison with the corresponding single species counterpart; (2) identification of the non-cytotoxic BSs concentrations with a significant inhibitory activity against single species biofilm formation; (3) evaluation of the anti-biofilm and antimicrobial activity of BSs at the non-cytotoxic concentrations against polymicrobial cultures, in co-incubation; (4) evaluation of the anti-biofilm and antimicrobial activity of silicone discs coated with BSs against polymicrobial cultures; (5) assessment of cells surface hydrophobicity and membrane permeability changes induced by BSs in soluble form; (6) observation of the dual-species biofilm micro-structure on BSs-coated silicone discs; and (7) preliminary assessment of BSs-coated silicone discs biocompatibility.

### Strains

The rhamnolipid-producer strain *Pseudomonas aeruginosa* 89, a clinical isolate from a patient with cystic fibrosis, was cultured from frozen stocks onto Tryptic Soy Agar (TSA, Scharlab, Barcelona, ES) at 37°C for 18–20 h. The lipopeptide-producer strain *Bacillus subtilis* AC7, from the inside of stems of *Robinia pseudoacacia*, was cultured from frozen stocks onto Luria Bertani agar (LBA, Sigma–Aldrich, St. Louis, MO, United States) at 28°C for 18–20 h. The sophorolipid-producer strain *Candida bombicola* ATCC 22214, obtained from the American Type Culture Collection (ATCC, Manassas, VA, United States), was cultured from frozen stocks onto Sabouraud dextrose agar (SDA, Scharlab, Barcelona, ES) at 25°C for 18–20 h. All the biofilm-producer strains used in this study were obtained from the ATCC (Manassas, VA, United States). *C. albicans* ATCC 10231, *S. aureus* ATCC 25923, *S. aureus* ATCC 6538, and *S. epidermidis* ATCC 35984 were cultured from frozen stocks onto SDA and TSA plates, respectively, and incubated overnight at 37°C.

### Biosurfactants Production and Extraction

Lipopeptide AC7BS and rhamnolipid R89BS were produced and extracted as described by Ceresa et al. (2018, 2019b). Briefly, a loop of *B. subtilis* AC7 from a LBA overnight culture was grown in 20 ml of LB broth at 28°C for 4 h at 140 r/min. Afterward, 2 ml of this culture was used to inoculate 500 ml of LB broth and was incubated at 28°C for 24 h at 140 r/min. A loop of *P. aeruginosa* 89 from a TSA overnight culture was grown in 40 ml of Nutrient Broth II (Sifin Diagnostics GmbH, Berlin, DE) for 4 h at 37°C at 140 r/min. Afterward, 8 ml of this culture was added to 400 ml of Siegmund–Wagner medium and incubated at 37°C for 5 days at 120 r/min. Cell-free supernatants were acidified to pH 2.2 with 6 M HCl (AC7BS) or 6 M H_2_SO_4_ (R89BS) and stored overnight at 4°C. Lipopeptide AC7BS and rhamnolipid R89BS were extracted three times with 167 ml ethyl acetate:methanol (4:1) or 134 ml ethyl acetate (Sigma–Aldrich, St. Louis, MO, United States), respectively. Organic phases were anhydrified, filtrated, and vacuum-dried. BSs were recovered by dissolution in acetone (Sigma–Aldrich, St. Louis, MO, United States) and collected in glass tubes. Acetone was, then, evaporated and BSs were weighted.

Sophorolipid SL18 was obtained from a fed batch cultivation of *C. bombicola* ATCC 22214, according to Ceresa et al. (2020). Briefly, the cells (10% v/v) were grown in 2 l of Glucose Yeast Urea (GYU) medium [100 g/l glucose (Sigma–Aldrich, St. Louis, MO, United States), 10 g/l yeast extract (Sigma–Aldrich, St. Louis, MO, United States), and 1 g/l urea (Sigma–Aldrich, St. Louis, MO, United States)]. Oleic acid (99%, Sigma–Aldrich, St. Louis, MO, United States) was supplemented as a feeding source at a concentration of 20% to generate lactonic congeners. Fermentation was performed for 8 days at 200 r/min and 30°C. Sophorolipid SL18 were, then, extracted twice with ethyl acetate (1:1 extract ratio) (Sigma–Aldrich, St. Louis, MO, United States) and partially purified by three washings with hexane (Sigma–Aldrich, St. Louis, MO, United States) to remove residual fatty acids.

At the end of the extraction process, the presence, the purity, and the composition of the three BSs were confirmed by ESI/MS analysis as previously described (Ceresa et al., 2016, 2019b, 2020). All the following biological tests and microscopy analyses were performed using the same batch of production for each BS. BSs were dissolved in Phosphate Buffer Solution pH 7.4 (PBS) at the different concentrations of use. The solutions were filtered through a 0.2 μm filter and stored at room temperature.

### Silicone Cleaning and Sterilization

Silicone-elastomeric discs (SEDs—0.8 cm in diameter and 1.5 mm in thickness) were cut from medical-grade silicone sheets (TECNOEXTR s.r.l, Palazzolo sull’Oglio, IT) and prepared as described in Ceresa et al. (2015). Briefly, discs were cleaned with a 1.4% (v/v) RBS^TM^ 50 solution (Sigma–Aldrich, St. Louis, MO, United States), sonicated for 5 min at 60 kHz, and rinsed twice in Milli-Q water. Silicone was, then, dipped in MeOH (99%, Sigma–Aldrich, St. Louis, MO, United States), sonicated, and rinsed as previously described. Afterward, SEDs were autoclaved, dried, and moved aseptically into 48-well plates.

### Anti-biofilm Assays

Mono- and dual-species biofilm formation: fungal and bacterial cells were suspended in Roswell Park Memorial Institute (RPMI) 1640 (Sigma–Aldrich, St. Louis, MO, United States) buffered with MOPS (Sigma–Aldrich, St. Louis, MO, United States) and supplemented with 2% Glucose (Biolife, Monza, IT), pH 7.0 (RPMI +2%G). Cell density was adjusted up to 10^6^ and 10^7^ colony forming unit (CFU)/ml for *C. albicans* and *Staphylococcus* spp. respectively. Polystyrene was used as a substrate for the growth of biofilms in co-incubation assays and silicone was used for the growth of biofilms in the coating assays. Surfaces were inoculated with 0.5 ml of the suspension and incubated at 37°C in static conditions up to 72 h; growth medium was removed and replaced with fresh RPMI +2%G every 24 h. Blank polystyrene and silicone control surfaces (without biofilm) were also included in the experimental setting. The ability of microbial strains to form polymicrobial biofilms, compared to the mono-species ones, was evaluated by the determination of biofilm biomass and metabolic activity of sessile cells as described below. All experiments were carried out in quadruplicate and repeated two times.

Co-incubation conditions (BSs in soluble form in polystyrene plates): in order to determine the minimum non-cytotoxic concentration of BSs (Ceresa et al., 2018, 2019b) able to inhibit single-species biofilm formation on polystyrene by at least 80%, increasing concentrations of lipopeptide AC7BS (0.125–0.5 mg/ml) and rhamnolipid R89BS (0.0125–0.05 mg/ml) were tested. The wells were filled with 50 μl of 10× BSs solutions (treated samples) or with an equal volume of PBS (control samples) and 0.5 ml of single-species suspensions of *C. albicans* (10^6^ CFU/ml) and *Staphylococcus* spp. (10^7^ CFU/ml) in RPMI +2%G. The 48-well plates were incubated for 24 h at 37°C. The effect was evaluated in terms of biofilm biomass reduction as described below. All experiments were carried out in quadruplicate and repeated twice.

Subsequently, the selected concentrations of BSs were tested against dual-species biofilm formation. The bottom of the wells was covered with 50 μl of the selected 10× BSs solutions (treated samples) or with an equal volume of PBS (control samples). Then, 0.5 ml of the dual-species suspensions of *C. albicans* (10^6^ CFU/ml) and *Staphylococcus* spp. (10^7^ CFU/ml) in RPMI +2%G were added to each well. The plates were incubated up to 72 h at 37°C. Growth medium was removed and replaced with fresh RPMI +2%G supplemented with 10× BSs solutions (treated samples) or with an equal volume of PBS (control samples) every 24 h. Blank surfaces (without biofilm) were also included in the experimental setting. The ability of microbial surfactants to inhibit dual-species biofilm formation in co-incubation conditions was evaluated by the determination of biofilm biomass and metabolic activity of sessile and planktonic cells as described below. Experiments were performed in quadruplicate and repeated twice.

Coating conditions: the treatment of silicone surfaces was carried out in 48-well plates by immersing SEDs in BS solutions (rhamnolipid R89BS: 2 mg/ml; lipopeptide AC7BS: 2 mg/ml; sophorolipid SL18: 8 mg/ml) at 37°C for 24 h at 180 r/min. These solutions were chosen as previously optimized in Ceresa et al. (2016; 2019b; 2020). Afterward, discs were moved into new plates and dried before use. Five hundred microliters of the dual-species suspensions (*C. albicans* at the concentration of 10^6^ CFU/ml and *Staphylococcus* spp. at the concentration of 10^7^ CFU/ml) in RPMI +2%G were added to each well. SEDs were incubated up to 72 h at 37°C. Every 24 h, discs were moved into fresh media. Blank surfaces (without biofilm) were also included. The anti-biofilm activity of BSs was evaluated at 24, 48, and 72 h by the determination of biofilm biomass, metabolic activity of sessile and planktonic cells, and viable cell counting as described below. Experiments were performed in quadruplicate and repeated twice.

#### Biofilm Biomass

The determination of the total biomass was carried out by crystal violet staining according to Ceresa et al. (2019b), with minor changes. Briefly, biofilms were washed twice and stained with 0.5 ml of the CV solution (0.1%) for 10 min. After the removal of the excess of dye, CV was dissolved with 0.5 ml of acetic acid (33% in water). Absorbance of the solutions was measured at 570 nm (A_570_) (Victor3V^TM^, Perkin Elmer, Italy), data were normalized to blank surfaces (background), and percentages of inhibition were calculated using the following formula:

(1) $$\left(1-\frac{{\text{A}}_{\text{treat}}}{{\text{A}}_{\text{CTRL}}}\right)\times 100$$

where

A_treat_: absorbance of treated samples

A_CTRL_: absorbance of controls.

#### Biofilm Metabolic Activity

The determination of biofilm metabolic activity was carried out by means of the colorimetric MTT assay according to Ceresa et al. (2019b), with minor changes. Briefly, biofilms were washed twice and, then, dipped in 0.5 ml of MTT working solution [0.075% MTT (Scharlab, Barcelona, ES) solution supplemented with 0.1% glucose (Biolife, Monza, IT) and 10 μM menadione (Sigma–Aldrich, St. Louis, MO, United States)]. After 30 min of incubation at 37°C in static conditions, MTT solution was removed and formazan crystals formed by metabolic active cells within biofilms were dissolved with 0.5 ml of the lysis solution [dimethyl sulfoxide (DMSO)/0.1 M glycine buffer (pH 10.2) solution (7:1)]. The A_570_ of the solutions was measured, data were normalized to background, and percentages of inhibition were calculated according to formula (1).

#### Planktonic Cells Metabolic Activity

The metabolic activity of planktonic cells in supernatants was evaluated by the MTT assay according to Ceresa et al. (2019b), with minor changes. Briefly, growth media and washing solutions, from each treated and untreated surface, were collected after 24, 48, and 72 h. Microbial cells were harvested by centrifugation at 17,000 r/min for 15 min and incubated in 0.5 ml of the MTT working solution for 30 min. Afterward, cells were collected by centrifugation at 17,000 r/min for 15 min, MTT solution was removed, and formazan crystals were dissolved with 0.5 ml of the lysis solution. The A_570_ of the solutions was measured and data were normalized to background.

### Quantification of Viable Sessile Cells

The number of microbial cells forming the multi-species biofilms was determined by the spread plate method as described in Ceresa et al. (2015). Briefly, after two washings, biofilms were detached from silicone surfaces and broke up by four cycles of sonication (30 s) and stirring (30 s). The obtained suspensions were serially diluted in PBS and seeded both on Mannitol Salt Agar (MSA, Scharlab, Barcelona, ES) plates, selective for staphylococcal species, and on Sabouraud Chloramphenicol Agar (SCA, Scharlab, Barcelona, ES) plates, selective for fungal species. After 24 h at 37°C, the colonies were counted and the number of *C. albicans*, *S. aureus*, *or S. epidermidis* cells within the polymicrobial biofilm was quantified.

### Anti-adhesive Assay

Silicone-elastomeric discs surface coating with the BSs was carried out as described by Papa et al. (2015), with minor changes. Briefly, a volume of 20 μl of BS solutions (rhamnolipid R89BS: 2 mg/ml; lipopeptide AC7BS: 2 mg/ml; sophorolipid SL18: 8 mg/ml) or 20 μl of PBS as control, were deposited on the silicone surfaces. SEDs were then placed under laminar flow to allow complete drying and, subsequently, moved into 48-well plates. The discs were filled with 0.5 ml of the dual-species suspensions (*C. albicans* at the concentration of 10^6^ CFU/ml and *Staphylococcus* spp. at the concentration of 10^7^ CFU/ml in RPMI +2%G) and incubated at 37°C for 4 h. The quantification of cells attached on SEDs was carried out using crystal violet staining as reported in Section “Biofilm Biomass.” All experiments were carried out in quadruplicate and repeated twice.

### Cell Surface Hydrophobicity and Membrane Permeability Changes by BSs in Soluble Form

#### Cell Surface Hydrophobicity

Bacterial and fungal suspensions were prepared in PBS to obtain an optical density (OD) at 600 nm, respectively, of 0.5 and 0.4 and treated with BSs (final concentration R89BS—0.05 mg/ml, AC7BS—0.5 mg/ml) at 37°C for 1 h at 150 r/min. Untreated suspensions were taken as control. Cell hydrophobicity was measured by microbial adherence to hexadecane (Scharlab, Barcelona, ES) according to Rosenberg et al. (1980). Microbial cells were collected by centrifugation at 4000 r/min for 15 min and resuspended in PUM Buffer, pH 7.1 (22.2 g K_2_HPO_4_⋅3H_2_0, 7.26 g KH_2_PO_4_, 1.8 g urea, 0.2 g MgSO_4_⋅7H_2_0 and distilled water to 1 l). One milliliter of hexadecane was mixed to 4 ml of cell suspensions in a glass tube at high speed for 2 min and equilibrated for 10 min. Afterward, the ODs of the initial cell suspensions and aqueous phases were measured at 550 nm (Genova Plus, Jenway, United Kingdom) and cell hydrophobicity was calculated using the following formula:

(2) $$\left(1-\frac{{\text{OD}}_{\mathrm{aqueous}\mathrm{phase}}}{{\text{OD}}_{\mathrm{initial}\mathrm{cell}\mathrm{suspension}}}\right)\times 100$$

#### Cell Membrane Permeability

Bacterial and fungal suspensions were prepared in PBS to obtain an OD at 600 nm, respectively, of 0.5 and 0.4 and treated with BSs (final concentration: R89BS—0.05 mg/ml, AC7BS—0.5 mg/ml) at 37°C for 1 h at 150 r/min. Untreated suspensions were taken as control. Cell membrane permeability was evaluated by checking crystal violet enhanced penetration. Cells were collected by centrifugation at 4000 r/min for 15 min and resuspended in PBS containing crystal violet (10 μg/ml) and incubated at 37°C at 150 r/min for 20 min. Afterward, cells were collected by centrifugation at 4000 r/min for 15 min and the absorbance (A) of the solutions was measured at 590 nm (Genova Plus, Jenway, United Kingdom). The percentage of crystal violet uptake was estimated using the following formula:

(3) $$\left(\frac{{\text{A}}_{\text{sample}}}{{\text{A}}_{\mathrm{initial}\mathrm{crystal}\mathrm{violet}\mathrm{solution}}}\right)\times 100$$

Experiments were performed in triplicate and repeated three times.

### Biofilm Architecture

The micromorphology and architecture of multi-species biofilm on SEDs was visualized using the scanning electron microscope (SEM) Quanta 200F FEG (Fei, Eindhoven, Netherlands) in high-vacuum mode. Samples were prepared for SEM imaging according to Ceresa et al. (2019b), with minor changes. Briefly, after two washings, biofilms were fixed in 2.5% glutaraldehyde, washed twice in distilled water, dehydrated in a graded ethanol series, and coated with a 10 nm layer of gold with a sputter coater (Emitech K500X, Quorum Technologies, Laughton, United Kingdom). A set of representative images at a magnification of 500×, 1000×, 2000×, and 4000× were obtained from untreated (controls) and pre-coated SEDs with rhamnolipid R89BS or sophorolipid SL18 and incubated with either *C. albicans–S. epidermidis* or *C. albicans–S. aureus* at 24, 48, and 72 h. Secondary electron signal was collected to investigate structural details of microbial cells and extracellular matrix on the biofilm. The primary beam energy was set to 5 keV to minimize damage to the organic structures. Possible artifacts due to the sample preparation process were considered according to indications provided by Hrubanova et al. (2018) and previous experience performed in imaging microbial biofilm formed *in vitro* on medical devices (Tessarolo et al., 2007; Signoretto et al., 2013).

### Biocompatibility of BS-Coated Silicone Discs

The *in vitro* biocompatibility of BSs-coated discs was evaluated in 48-well plates by the MTT assay (Ceresa et al., 2019a). Spontaneously immortalized human skin keratinocyte—HaCaT cells (10^4^ cells/well) were seeded in Dulbecco’s Modified Eagle’s Medium (DMEM) high glucose (EuroClone, Milan, IT) supplemented with 4% FBS (EuroClone, Milan, IT), L-glutamine 200 nM (EuroClone, Italy) and 1% Pen/Strep (EuroClone, Milan, IT), and incubated at 37°C in 5% CO_2_. After 24 h, growth medium was removed and replaced with the eluates obtained from BSs-coated SEDs after static release at 37°C for 24 h. Negative control consisted in cells treated with 0.5% Triton X, whereas positive control was represented by cells w/o any treatment. Fifty microliters of the MTT solution (5 mg/ml) was added into each well. Plates were then incubated for 24 and 72 h at 37°C. Formazan crystals were dissolved with 200 μl of 0.05 M HCl/isopropanol (50:1) and A570 was measured at the two time points. The percentage of cell viability was estimated using the following formula:

(4) $$\left(\frac{{\text{A}}_{\text{sample}}}{{\text{A}}_{\text{CTRL}+}}\right)\times 100$$

where

A_sample_: absorbance of BSs or CTRL—samples

A_CTRL__+_: absorbance of positive controls.

Experiments were performed in triplicate and repeated twice.

### Data Analysis and Statistics

All analyses and graphics were performed using the statistical program R, 3.6.2 (R Core Team, 2019). One-way ANOVA was applied to compare mono- and dual-species biofilms. Two-way ANOVA followed by Tukey *post hoc* test was used to investigate the anti-biofilm activity of BSs on dual-species biofilms and the metabolic activity of planktonic cells. To estimate log_10_ CFU/disk from colony counts, the R package dupiR was used (Comoglio et al., 2013). Differences in the percentage composition of dual-species biofilms were investigated by two-sample *t*-test for equality of proportions with continuity correction. Two-sample *t*-test was performed to evaluate the significance of data in hydrophobicity and membrane permeability assays. One-way ANOVA followed by Tukey *post hoc* test was performed to evaluate the significance of data in the biocompatibility assay in comparison to positive and negative controls. Differences were considered statistically significant at *p* < 0.05.

## Results

### Biosurfactants Anti-biofilm and Anti-adhesive Activity

**FIGURE 1:**
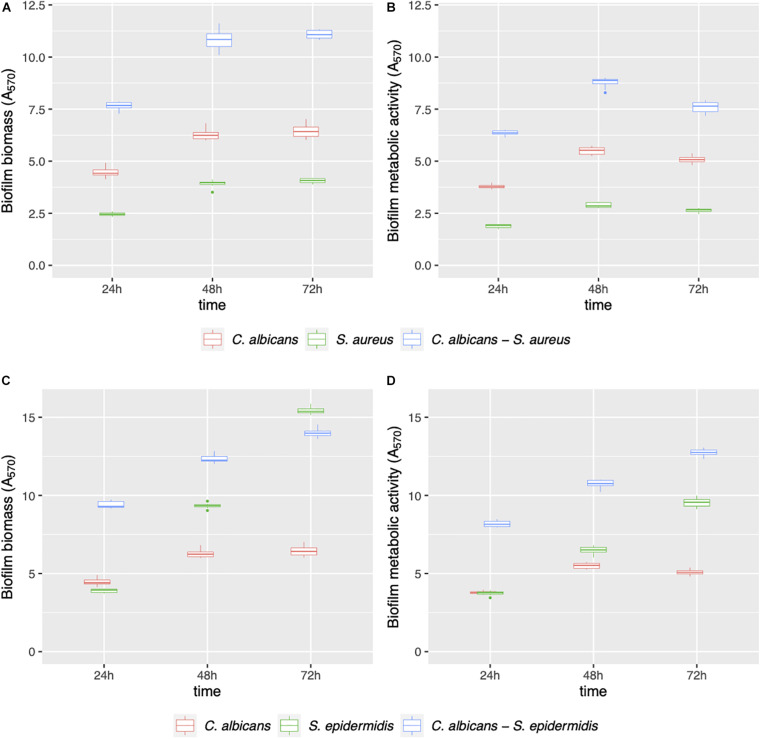
Mono- and dual-species *C. albicans*–*Staphylococcus* spp. biofilm formation on polystyrene. The ability of microbial strains to form biofilms was evaluated at 24, 48, and 72 h by biofilm biomass **(A,C)** and biofilm metabolic activity **(B,D)** quantification.

**FIGURE 2:**
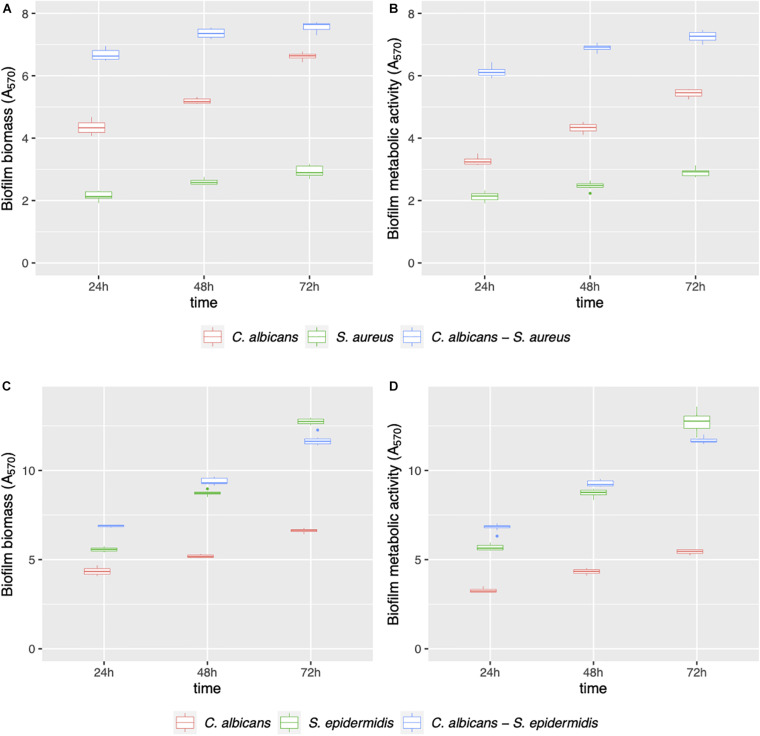
Mono- and dual-species *C. albicans*–*Staphylococcus* spp. biofilm formation on silicone discs. Biofilms were evaluated in terms of biofilm biomass **(A,C)** and biofilm metabolic activity **(B,D)** at 24, 48, and 72 h.

Mono- and dual-species biofilms of *C. albicans*–*Staphylococcus* spp. were grown on polystyrene (Figure 1) and on silicone surfaces (Figure 2) up to 72 h. Biofilm biomass and biofilm metabolic activity were quantified at 24, 48, and 72 h. *C. albicans*–*S. aureus* biomass and metabolic activity were higher than those of the two individual species both on polystyrene and on silicone at all incubation times (*p* < 0.001). Concerning *C. albicans*–*S. epidermidis*, biomass and metabolic activity were always higher than those observed for the individual species, on both surfaces, up to 48 h (*p* < 0.001). On the contrary, at 72 h, biomass and metabolic activity of dual-species biofilms were lower than those observed for *S. epidermidis* biofilms, with the exception of metabolic activity on polystyrene (*p* < 0.001).

**FIGURE 3:**
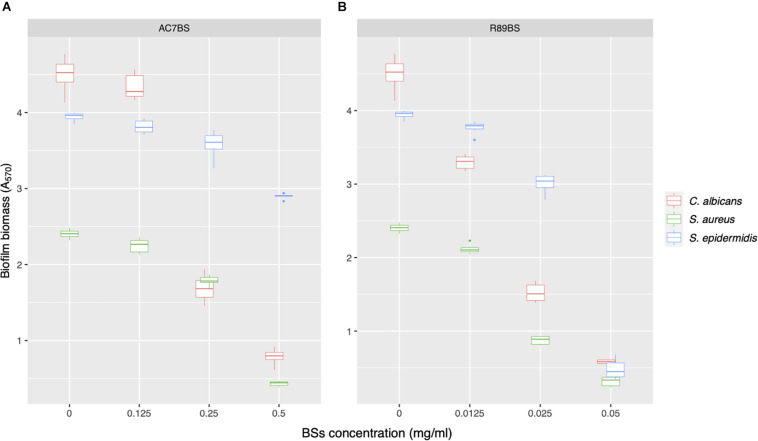
Activity of BSs in soluble form on *C. albicans*, *S. aureus*, and *S. epidermidis* biofilm formation. The anti-biofilm activity of AC7BS **(A)** and R89BS **(B)** was evaluated in co-incubation conditions by the determination of biofilm biomass.

AC7BS and R89BS were selected to perform the co-incubation assays as they were previously reported as not cytotoxic versus MRC5 cells monolayers at the concentrations active against biofilm (Ceresa et al., 2018, 2019b). On the contrary, SL-18 was not included in this assay since it was previously detected as cytotoxic when used in soluble form at concentrations active against *C. albicans* and *S. aureus* single species biofilms (Ceresa et al., 2020). Increasing concentrations of lipopeptide AC7BS (0.125–0.5 mg/ml) and rhamnolipid R89BS (0.0125–0.05 mg/ml) were tested and the minimum concentration of BSs that counteracted single-species biofilms formation on polystyrene by at least 80% (inhibition level threshold) was identified (Figure 3). In general, the formation of *C. albicans* and *S. aureus* biofilms was reduced in a concentration-dependent manner by the two BSs while the formation of *S. epidermidis* biofilm was effectively inhibited (≥80%) only by rhamnolipid R89BS (Figure 3A). In particular, the three mono-species biofilms were inhibited by about 87% by rhamnolipid R89BS at a concentration of 0.05 mg/ml. Concerning lipopeptide AC7BS (Figure 3B), the threshold inhibition level was reached at a concentration of 0.5 mg/ml only for *C. albicans* and *S. aureus* biofilms (82%) but not for *S. epidermidis*, which had a maximum inhibition of only 27%. For this reason, this BS was excluded from the subsequent anti-biofilm assays against *C. albicans*–*S. epidermidis* dual-species biofilms.

**FIGURE 4:**
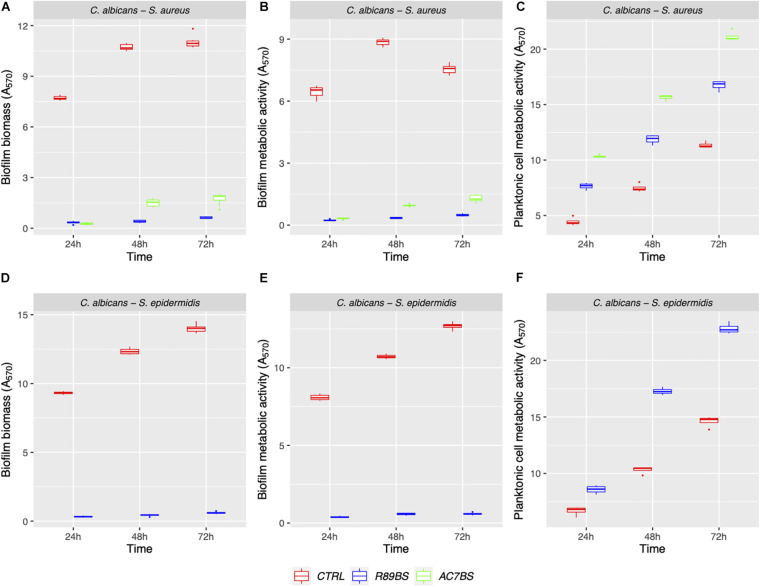
Anti-biofilm activity of BSs in soluble form on *C. albicans*–*S. aureus* and *C. albicans*–*S. epidermidis* biofilm formation. The anti-biofilm activity of BSs was evaluated in co-incubation conditions in terms of biofilm biomass **(A,D)** and biofilm metabolic activity **(B,E)**. The viability of planktonic cells **(C,F)** was measured by the metabolic activity assay.

The boxplot in Figure 4 shows the effect of rhamnolipid R89BS and lipopeptide AC7BS against *C. albicans*–*S. aureus* and of R89BS against *C. albicans*–*S. epidermidis* biofilm formation up to 72 h. In general, as confirmed by two-way ANOVA and Tukey *post hoc* test, for each co-culture, biofilm formation on polystyrene was significantly dependent on BSs treatment (*p* < 0.001) and incubation time (*p* < 0.001). Regardless of the two strain combinations involved in the dual-species biofilm development, biofilm biomass (Figures 4A,D) and metabolic activity (Figures 4B,E) were equally inhibited by the BSs. The anti-biofilm activity of rhamnolipid R89BS was stable up to 72 h, while, for lipopeptide AC7BS, a slight reduction was detected between 24 and 72 h. In particular, as observed in Table 1, rhamnolipid R89BS proved to be the most effective BS for the inhibition of *C. albicans*–*S. aureus* biofilm growth and an excellent agent for the prevention of *C. albicans*–*S. epidermidis* biofilm formation, with mean percentages of reduction of 94% (*C. albicans*–*S. aureus*) and 95% (*C. albicans*–*S. epidermidis*), after 72 h co-incubation. In addition, to define whether part of the observed effect was the result of an antimicrobial activity of the BSs, the metabolic activity of planktonic cells in the supernatants was assessed (Figures 4C,F). The absorbance values of the cell supernatants co-incubated with BSs were significantly higher in comparison to the controls (*p <* 0.001) suggesting that, in the treated wells, cells existed in a planktonic state rather than by forming a biofilm. However, in the case of *C. albicans*–*S. aureus* co-cultures, the lower absorbance values observed in the rhamnolipid R89BS-treated wells compared to those of the lipopeptide AC7BS-treated wells (*p <* 0.001), suggested an antimicrobial activity of the rhamnolipid (Figure 4C).

**TABLE 1:** Inhibition percentages of dual-species *C. albicans*–*S. aureus* and *C. albicans*–*S. epidermidis* biofilm formation on polystyrene in co-incubation assays. The anti-biofilm activity of BSs in soluble form was detected by CV (biofilm biomass) and MTT assays (biofilm metabolic activity).

Strains	Assessment	Time (h)	Treatment	
			AC7BS (%)	R89BS (%)
*C–Sa*	Biofilm biomass	24	96.4	95.6
		48	85.8	96.2
		72	84.3	94.3
	Biofilm metabolic activity	24	95.0	96.4
		48	89.2	96.1
		72	83.0	93.5
C–*Se*	Biofilm biomass	24	−	96.5
		48	−	96.5
		72	−	95.7
	Biofilm metabolic activity	24	−	95.1
		48	−	94.6
		72	−	95.3

*C–Sa, C. albicans–S. aureus; C–Se, C. albicans–S. epidermidis; −, not evaluated.*

**FIGURE 5:**
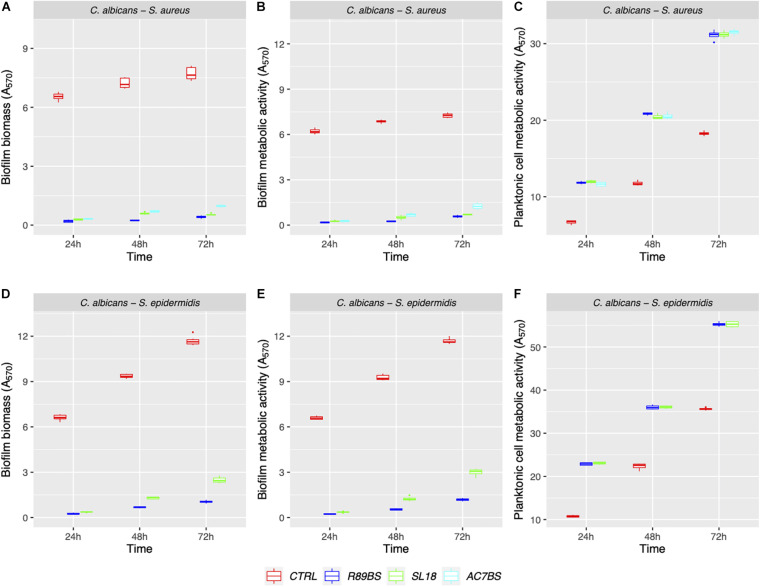
Anti-biofilm activity of the BSs coated silicone discs. The efficacy of the surface treatments was evaluated in terms of biofilm biomass **(A,D)** and biofilm metabolic activity **(B,E)**. The viability of planktonic cells was measured by the metabolic activity assay **(C,F)**.

The results of BSs pre-coating on SEDs further confirmed the inhibitory activity of these natural microbial molecules against the formation of *C. albicans*–*S. aureus* (rhamnolipid R89BS, lipopeptide AC7BS, sophorolipid SL18) and *C. albicans*–*S. epidermidis* (rhamnolipid R89BS, sophorolipid SL18) mixed biofilms (Figure 5). As previously observed in co-incubation conditions, for each co-culture, the development of biofilms on silicone surfaces was significantly dependent on BSs treatment (*p* < 0.001) and incubation time (*p* < 0.001). Biofilm biomass (Figures 5A,D) and metabolic activity (Figures 5B,E) were equally inhibited on all BSs-coated discs (SEDs). Concerning the dual-species biofilms of *C. albicans*–*S. aureus*, the anti-biofilm activity of rhamnolipid R89BS- and sophorolipid SL18-coated SEDs was stable up to 72 h, while a slight reduction of the efficacy of lipopeptide AC7BS-coated SEDs was observed during this time. The anti-biofilm activity of rhamnolipid R89BS coating was stable also for *C. albicans*–*S. epidermidis* mixed biofilms, while that of sophorolipid SL18-coated SEDs slightly decreased over time. In general, starting from 48 h of incubation, the surface treatment of silicone with rhamnolipid R89BS proved to be the most effective in counteracting the growth of both polymicrobial biofilms. In particular, after 72 h, average inhibitions of 93 and 90% against *C. albicans*–*S. aureus* and *C. albicans*–*S. epidermidis* biofilms were found, respectively. Table 2 shows the percentages of biomass and metabolic activity inhibition at the different time-points. To exclude that the observed activity was due to an antimicrobial action of the BSs, the metabolic activity of the planktonic cells in the wells was evaluated (Figures 5C,F). As observed in co-incubation conditions, the absorbance values of the supernatants of the BSs-coated silicone discs were significantly higher than those obtained for the corresponding controls (*p* < 0.001). Conversely, no significant variations were found between the absorbance values of planktonic cells recorded for the different BSs treatments (*p* > 0.05).

**TABLE 2:** Inhibition percentages of dual-species *C. albicans–S. aureus* and *C. albicans–S. epidermidis* biofilm formation on BSs treated silicone discs by CV (biofilm biomass) and MTT assays (biofilm metabolic activity).

Strains	Assessment	Time (h)	Treatment		
			AC7BS (%)	R89BS (%)	SL18 (%)
*C–Sa*	Biofilm biomass	24	95.1	96.9	95.6
		48	90.2	96.7	91.8
		72	87.3	94.6	93.1
	Biofilm metabolic activity	24	95.6	97.2	95.8
		48	90.0	96.3	92.6
		72	82.8	92.0	90.3
C–*Se*	Biofilm biomass	24	−	96.1	94.4
		48	−	97.7	86.1
		72	−	91.0	78.8
	Biofilm metabolic activity	24	−	96.5	94.5
		48	−	94.1	86.7
		72	−	89.8	74.4

*C–Sa, C. albicans–S. aureus; C–Se, C. albicans–S. epidermidis; −, not evaluated.*

In addition, to evaluate whether the tested BSs exhibited their anti-biofilm action in equal proportions on the single species forming the polymicrobial biofilms on the silicone surfaces, the number of cells of *C. albicans*, *S. aureus*, and *S. epidermidis* was determined on selective media for both control and coated discs (Supplementary Tables 1, 2) and the percentage compositions were calculated (Tables 3, 4). In general, at all incubation times, biofilms on the control discs mainly consisted of bacterial cells (98.6–99.8%) and only for a small percentage of the yeast cells (0.2–1.4%). In particular, the yeast cells were present in a major proportion in *C. albicans*–*S. aureus* biofilms (Table 3) than in *C. albicans*–*S. epidermidis* (Table 4). Interestingly, at 24 h, the presence of BSs on the silicone surface was associated with a significant increase in the yeast species percentage compared to that observed on the controls (*p <* 0.001). However, this value decreased over time and returned to levels similar to those observed for the controls.

**TABLE 3:** Percentage (%) composition of *C. albicans*–*S. aureus* dual-species biofilms on the surface of silicone discs.

Time (h)	Strains	Treatment			
		CTRL (%)	AC7BS (%)	R89BS (%)	SL18 (%)
24	*C. albicans*	1.4	4.5	4.9	4.0
	*S. aureus*	98.6	95.5	95.1	96.0
48	*C. albicans*	1.4	1.6	1.7	0.9
	*S. aureus*	98.6	98.4	98.3	99.1
72	*C. albicans*	1.3	1.4	0.3	0.5
	*S. aureus*	98.7	98.6	99.7	99.5

**TABLE 4:** Percentage (%) composition of *C. albicans*–*S. epidermidis* dual-species biofilms on the surface of silicone discs.

Time (h)	Strains	Treatment		
		CTRL (%)	R89BS (%)	SL18 (%)
24	*C. albicans*	0.2	1.0	0.6
	*S. epidermidis*	99.8	99.0	99.4
48	*C. albicans*	0.2	0.2	0.1
	*S. epidermidis*	99.8	99.8	99.9
72	*C. albicans*	0.2	0.2	0.2
	*S. epidermidis*	99.8	99.8	99.8

**FIGURE 6:**
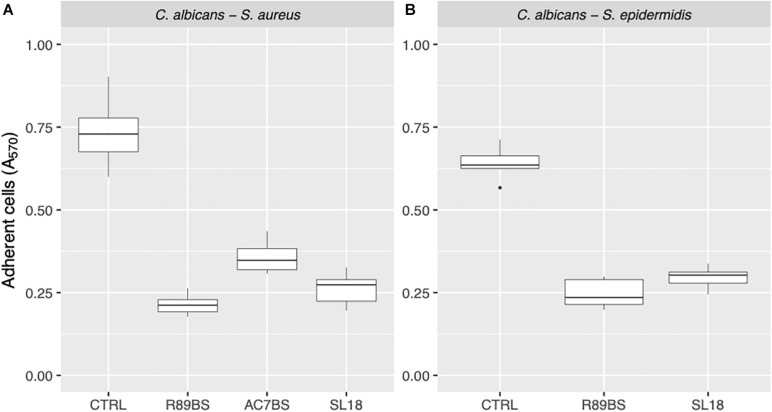
Anti-adhesive activity of the BSs coated silicone discs. The efficacy of the BSs surface treatment was evaluated in terms of *C. albicans*–*S. aureus* **(A)** and *C. albicans*–*S. epidermidis* **(B)** adherent cells after 4 h of incubation.

Finally, to assess the activity of BSs coating on SEDs on the early phases of biofilm formation (adhesion phase), the amount of *C. albicans*–*S. aureus* and *C. albicans*–*S. epidermidis* adherent cells was evaluated after 4 h incubation by means of the CV method (Figure 6). Similarly, to biofilm formation, microbial cells adhesion to silicone surfaces was also significantly dependent on BSs treatment (*p* < 0.001). Concerning *C. albicans*–*S. aureus*, the highest anti-adhesive activity was observed for rhamnolipid R89BS (71%), followed by sophorolipid SL18 (64%) and lipopeptide AC7BS (51%). As to *C. albicans*–*S. epidermidis*, rhamnolipid R89BS coating proved to be the most effective with an inhibition of adhesion of 62% while for sophorolipid SL18 showed a 54% inhibition.

### Cell Surface Hydrophobicity and Membrane Permeability Changes by BSs in Soluble Form

**FIGURE 7:**
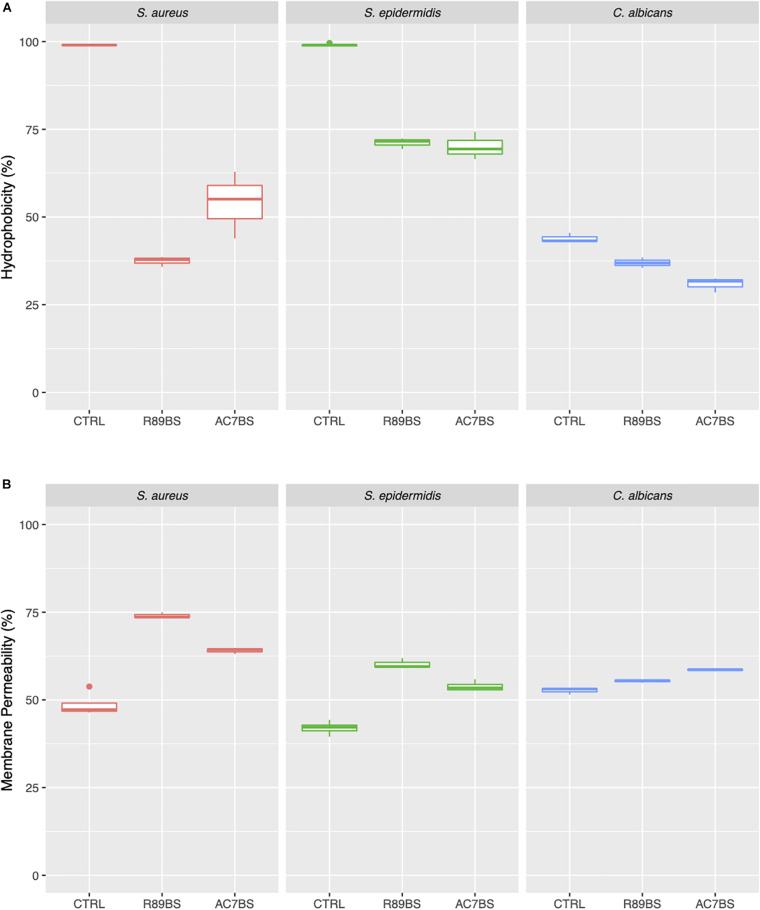
Changes in cell surface hydrophobicity and membrane permeability induced by soluble BSs. Cell surface hydrophobicity **(A)** and membrane permeability **(B)** of *C. albicans*, *S. aureus*, and *S. epidermidis* treated with BSs in soluble form (co-incubation conditions), compared to control samples.

In order to evaluate the possible effect of BSs on the tested opportunistic pathogens in co-incubation conditions, cell surface hydrophobicity (CSH) and membrane permeability were assessed. As shown in Figure 7A, after 1 h incubation with R89BS and AC7BS, CSH and membrane permeability of *C. albicans*, *S. aureus*, and *S. epidermidis* were different from the untreated controls. BSs treatment induced a significant modification of CSH for all the tested strains (*p* < 0.05). In particular, it resulted in a decrease of CSH both for bacterial and fungal cells. Bacterial CSH decreased from 99% (mean percentage for untreated control samples) to values ranging from 38 to 71% for BSs treated samples. Fungal CSH in comparison was reduced from 44% (mean percentage for control samples) to values ranging from 31 to 37% for BSs treated samples. The changes of CSH induced by BSs were related to the microbial strain: in particular, *S. aureus* was the most susceptible to cell hydrophobicity changes, followed by *S. epidermidis* and *C. albicans*. The treatment with BSs also altered cell membrane permeability for all the tested strains (*p* < 0.05) (Figure 7B). In particular, BSs induced an increase in crystal violet uptake by bacterial cells from 46% (mean percentage for untreated control samples) to values ranging from 54 to 74% and a slight increase in the case of fungal cells from 53% (mean percentage for untreated control samples) to values ranging from 56 to 59%. Again, the changes of membrane permeability induced by BSs were related to the microbial strain: in particular, *S. aureus* and *S. epidermidis* were more susceptible to BSs than *C. albicans*.

### Scanning Electron Microscopy of Multi-species Biofilms on SEDs

**FIGURE 8:**
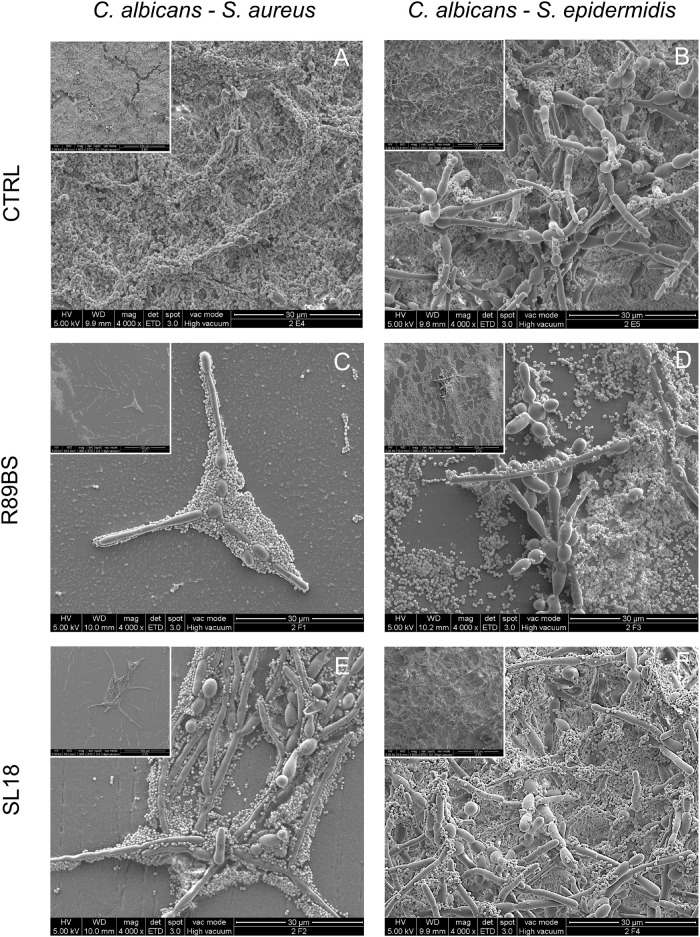
Scanning electron microscopy images of the dual-species biofilms formed on the silicone discs surface at 72 h. *C. albicans*–*S. aureus* on the left column and *C. albicans*–*S. epidermidis* on the right column. Different surface pre-coating treatments are presented: untreated controls (top row), rhamnolipid R89BS (middle raw), and sophorolipid SL18 (bottom row) treated discs. Insets present a lower magnification of the corresponding image to appreciate both macrostructural arrangement of the biofilm on the surface and micro-structural architecture of the two species in each biofilm sample. Original magnification: 4000x (1000x for the insets).

Scanning electron microscopic images presenting the features of the biofilm formed on untreated (controls) and pre-coated SEDs with rhamnolipid R89BS and sophorolipid SL18 at the longest incubation time are presented in Figure 8. The SEM investigation showed the presence of both fungal hyphae and coccoid bacteria adhering to the surface of all samples. In general, there was a more pronounced spatial correlation between *S. aureus* and *C. albicans* (Figures 8A,C,E) than between *S. epidermidis* and *C. albicans* (Figures 8B,D,F), irrespective of the surface treatment. More specifically, *S. aureus* was prevalently found in close contact with fungal hyphae, almost completely covering the *Candida* mycelium at 72 h in control SEDs (Figure 8A), while *S. epidermidis* never realized a full coating of the *C. albicans* structures (Figure 8B) and was more prone to adhere directly to the SEDs surface (Figure 8D).

In agreement with the results obtained from quantitative tests of biofilm biomass performed on corresponding samples, untreated controls showed a well-structured dual-species biofilm at each time-points, having a more three-dimensional arrangement and a thicker appearance at longer incubation times (Figures 8A,B). A similar increasing trend in the number of cells at the surface was also observed for rhamnolipid R89BS and sophorolipid SL18 pre-coated SEDs. However, the number of microbial cells at the treated surface was drastically reduced in respect of controls and the large majority of the sample surface was free of cells or presented small clusters with few three-dimensional microbial aggregates at 24 and 48 h (Supplementary Figures S1, S2). This was also the case for *S. aureus* and *C. albicans* dual-species biofilm at 72 h (Figures 8C,E), but not for *S. epidermidis* and *C. albicans* dual-species biofilm where only a minor portion of the treated surface was free of cells (Figure 8D) or mainly fully covered (Figure 8F). At 72 h, some differences in *C. albicans*–*S. epidermidis* biofilms were also noted between rhamnolipid R89BS and sophorolipid SL18 pre-coated discs, with the latter presenting areas showing a mature biofilm (Figure 8F), although less structured and thick than the controls (Figure 8B). Rhamnolipid R89BS pre-coated SEDs showed only minor portions of the disc surface still free of microorganisms (Figure 8D).

### Cytotoxicity of BSs-Coated Silicone Discs

No cytotoxic effect was detected on spontaneously immortalized human skin keratinocyte when exposed for 24 h to the BSs-coated SEDs eluates obtained from static release conditions. In fact, at this time-point, the viability of HaCat cells was comparable to positive controls (*p >* 0.05), independently to the type of BS involved in the coating procedure (Supplementary Figure S3A). After 72 h of cells exposure to the eluates, cell viability slightly decreased to values ranging from 92% for SL18 to 78% for R89BS (Supplementary Figure S3B) but was always above the limit (70%) according to the ISO 10993-5 standard (Xian, 2009; Rodríguez-López et al., 2020).

## Discussion

Microbial colonization and biofilm formation on medical devices represent one of the major challenges in infection control (Percival et al., 2015; Wang et al., 2017; Khatoon et al., 2018). Biofilms protect microbial cells from antimicrobials and the immune system of hosts and, in most cases, this may lead to the dysfunction of the devices and eventual removal (Percival et al., 2015; Khatoon et al., 2018; Sharma et al., 2019).

Nowadays, *C. albicans* is considered as the prevailing fungal pathogen responsible for severe hospital-acquired infections and has also been reported to form polymicrobial biofilms in coexistence with bacterial species such as *S. aureus*, *S. epidermidis*, *P. aeruginosa*, and *Streptococcus* spp. (Liu et al., 2019). These microbial pathogens are well known for their ability to form persistent biofilms on biotic and abiotic surfaces such as tissues, organs, and medical-devices, including dentures, voice prostheses, implants, endotracheal tubes, feeding tubes, and most frequently, catheters (Carolus et al., 2019; Liu et al., 2019).

The existence of these multi-species communities makes the challenge against biofilms even more complex because it does not only require effective antimicrobials against all pathogenic microorganisms present in the microbial community, but limits the effectiveness of to date-developed species-specific biofilm targeting strategies (Koo et al., 2017). Based on these considerations, numerous researchers have aimed their studies at identifying new and effective approaches to counteract polymicrobial biofilms, both in terms of inhibition of microbial adhesion and disruption of mature biofilms (Villa and Cappitelli, 2013). Among these, the use of new antimicrobial peptides has been suggested as a promising treatment against fungal/bacterial polymicrobial biofilms (Qu et al., 2016; de Alteriis et al., 2018; Gupta et al., 2019). Through a different mechanism of action, these molecules can cause microbial death, inhibit bacterial growth, and compromise biofilm formation and architecture. Weiland-Bräuer et al. (2019) identified, in the metagenome-derived bacterial quorum quenching (QQ), proteins QQ-5 and QQ-7 as an effective strategy to prevent *C. albicans* and *S. epidermidis* biofilm formation, by inhibiting *C. albicans* yeast-to-hyphae transition and inducing the expression of the *icaR* gene, thus repressing the synthesis of polysaccharide intercellular adhesion (PIA).

A further strategy currently proposed to treat *C. albicans*–S. *aureus* infections is based on combined therapies of existing antimicrobials or treatments with natural molecules, such as plant extracts or essential oils, alone or in combination with antibiotics or antifungals (Budzynska et al., 2017; Scaffaro et al., 2018; Tan et al., 2019).

In this scenario, BSs effectively appear to be promising new candidates for biofilm inhibition in the biomedical field due to their interesting antimicrobial, anti-adhesive properties (Banat et al., 2010; Rodrigues and Teixeira, 2010; Fracchia et al., 2019; Naughton et al., 2019). These molecules, in fact, are able to counteract effectively biofilms by decreasing microbial cells viability and by reducing microbial adhesion (Satpute et al., 2016; Fracchia et al., 2019; Paraszkiewicz et al., 2019; Naughton et al., 2019). When BSs bind to cell wall surface, they may form a film that changes the wettability and the surface energy of the cell leading to severe changes in its hydrophobicity and increasing its permeability by the release of LPS and the formation of transmembrane pores. When applied as coating agents, BSs interfere with microbial adhesion and limit biofilm formation altering the chemical and physical properties of the surfaces (e.g., reduction of roughness and hydrophobicity or increase of wettability) on which biofilms develop (Rodrigues et al., 2006; Quinn et al., 2013; Satpute et al., 2019).

In this study, we demonstrated the ability of different BSs to inhibit the formation of fungal and bacterial dual-species biofilms for up to three days in both co-incubation and pre-coating assays. One lipopeptide (AC7BS) and two glycolipids (R89BS and SL18) were tested. As observed by the positive ESI–MS analysis, AC7BS is composed of surfactin (98%) and fengycin (2%) homologs (Ceresa et al., 2016). Surfactin family members are represented by C13, C14, and C15 homologs whereas fengycin family members are represented by two main fengycin isoforms corresponding to C17 fengycin A and C17 fengycin B. The negative electrospray ionization (ESI) MS analysis of R89BS extract showed the presence of mono- (75%) and di- (25%) rhamnolipid homologs. Mono-rhamnolipid family members are represented by C10–C10, C8–C10, and C10–C12 homologs whereas di-rhamnolipid family members by C10–C8, C10–C10, and C10–C12 homologs (Ceresa et al., 2019b). Sophorolipid SL18 is a mixture of lactonic congeners (Ceresa et al., 2020). The fungal strain *C. albicans* and the bacterial strains *S. aureus* and *S. epidermidis* were selected as they represent a major cause of medical device associated infections due to their ability to adhere to biomaterials and form antimicrobial-resistant multispecies biofilms (Arciola et al., 2005; von Eiff et al., 2006; Sardi et al., 2013; Carolus et al., 2019).

The experiments reported in this study were carried out using culture media and growth conditions that support the reproducible development of well-structured dual-species biofilms, as demonstrated by the high values of biofilm biomass and cell metabolic activity detected for the controls and by SEM images of control biofilms. BSs were tested in solution or coated on silicone surfaces by physical absorption. Dual-species cultures were evaluated by the quantification of different parameters: biomass, metabolic activity, and number of viable cells. The microstructure of dual-species biofilms on treated and untreated silicone was also characterized by SEM analysis.

The lipopeptide AC7BS and the rhamnolipid R89B, tested in soluble form in co-incubation conditions, showed a significant inhibitory activity against the formation of dual-species biofilms of *C. albicans* and *Staphylococcus* spp. determining high levels of reduction, both in terms of total biomass and metabolic activity with inhibitions ranging from 84 to 96% at 72 h. These findings suggest a potential applicability of these BSs as components of pharmaceutical formulations, such as injectable scaffolds or hydrogels enriched with antimicrobial or anti-biofilm agents useful in wound healing. Similar conclusions were also reached by for better wound healing when microbial glycolipid BS-containing ointment was used as a transdermal substitute treatment process (Gupta et al., 2017).

Coating of silicone surfaces with lipopeptide AC7BS, rhamnolipid R89BS, and sophorolipid SL18 was as much beneficial, further confirming the inhibitory activity of these natural molecules against the formation of both dual-species biofilms, with percentages of inhibition at 72 h ranging from 77 to 93%. R89BS in particular was found to be the most effective BS in inhibiting both dual-species biofilms on both surfaces.

Both rhamnolipids and sophorolipids have also shown to be active against other multi-species cultures (Diaz De Rienzo et al., 2016). They observed using the BioFlux flow through conditions, that a combination of caprylic acid (0.01% v/v) and rhamnolipids (0.04% v/v) caused the disruption of single and mixed biofilms for *P. aeruginosa* and *S. aureus*. Biofilms were also efficiently dislodged by the combination of rhamnolipids (0.04% v/v) with sophorolipids (0.01% v/v). Interestingly, these authors observed that biofilm disruption of *S. aureus* and mixed cultures was caused by the anti-biofilm properties of BSs without affecting cell viability whereas for the *P. aeruginosa* biofilms, a high rate of killed cells was observed.

According to the studies conducted so far, in co-incubation conditions, the anti-biofilm activity of the tested BSs seemed to be related to a reduction of CSH and thus to a change in cells ability to adhere to the silicone surface (Elshikh et al., 2016). For rhamnolipid R89BS, the effect was also partly associated to its antibacterial activity on staphylococcal cells, as demonstrated in a previous work (Ceresa et al., 2019b) and confirmed in this study by the observed decrease of the planktonic cells metabolic activity and the increase in membrane permeability. On the contrary, an antifungal effect on *C. albicans* was not observed at the tested concentrations, indicating that the antimicrobial activity of the rhamnolipid is also microorganism dependent as observed by Diaz De Rienzo et al. (2016). Lipopeptide AC7BS was in general less effective in counteracting *S. epidermidis* biofilm formation as suggested by the fact that the inhibition level threshold of 80% was not reached when tested in co-incubation conditions against single species biofilm. In addition, CSH assays indicated that *S. epidermidis* was less susceptible to cell hydrophobicity changes induced by AC7BS compared to *S. aureus*. Moreover, *S. epidermidis* cell permeability was less affected in the presence of AC7BS in comparison to rhamnolipid R89BS. It may be hypothesized that the ability of this strain to produce a high amount of slime (Arciola et al., 2001; Williams and Bloebaum, 2010) might have interfered with the activity of AC7BS in the co-incubation conditions. Furthermore, AC7BS did not show any antimicrobial activity against *S. epidermidis* [Ceresa et al. Biosurfactant-based coatings inhibit fungal and bacterial biofilm on medical-grade silicone. TERMIS 2017. Davos, Switzerland, eCM Meeting Abstracts 2017, Collection 2; TERMIS EU (P821)] whereas, as far as R89BS is concerned, a MIC_99_ was detected at 120 μg/mL (Ceresa et al., 2019a) indicating a higher efficacy of the rhamnolipid against this strain compared to the lipopeptide.

When applied on silicone surfaces as coating agents, BSs effects were mostly related to their anti-adhesive properties. Surface physicochemical characterization of BSs-coated discs showed an increased level of wettability, i.e., a reduction of hydrophobicity (static contact angle for AC7BS-coated SEDs: 94.4° ± 10.0°; dynamic contact angle for R89BS-coated SEDs: 84.4° ± 2.2° (advancing) and 72.2° ± 2.5° (receding)), in comparison to control discs (112° ± 5°) (Ceresa et al., 2016, 2019b). In addition, concerning R89BS, the anti-biofilm effect in the pre-coating conditions was not due to an antimicrobial activity, as indicated by the fact that planktonic cells metabolic activity was similar to that observed in the presence of the other BSs, thus suggesting that the amount of R89 deposited on the silicone surface was largely below the biocidal concentration for staphylococci.

The impact of the BSs pre-coating in the formation of dual-species biofilm on the surface of medical grade silicon was also observed by SEM inspection. R89BS and SL18 almost completely prevented the attachment of the dual-species biofilms up to 48 h, and a clear reduction in the amount of biofilm with respect to controls was still evident at 72 h of incubation.

Differences in the cell arrangements and, more specifically, in the relative spatial distribution of cocci and yeasts were noted between *C albicans*–*S aureus* and *C. albicans*–*S. epidermidis*, whereas no influence on the inter-species spatial association was observed by comparing BS-coated and untreated silicone surfaces. A stronger association between the fungal and bacterial cells was observed in the *C albicans*–*S aureus* biofilm. As previously reported by Harriott and Noverr (2009), *S. aureus* mainly formed microcolonies on the surface of the biofilm, with *C. albicans* serving as the underlying scaffolding. Compared to *S. epidermidis*, *S. aureus* was not as effective in forming biofilms on abiotic surfaces, requiring precoating and supplementation of nutrients (Cassat et al., 2007). *C. albicans* was shown to play an essential role in producing extracellular matrix that facilitates *S. aureus* adhesion and sessile microcolonies formation (Harriott and Noverr, 2009). The *C. albicans*–*S. epidermidis* biofilm was characterized by a more open hyphal network and the association of *S. epidermidis* cells with the fungal structures was less marked. *S. epidermidis* cells were found beneath and above the yeast cells and hyphal layers and the Staphylococci cells were clearly adherent to both morphological forms of the fungus as previously reported by other authors (Adam et al., 2002). Anyway, the adhesion and microcolonies formations of *S. epidermidis* on the surface of the silicone samples were frequently noted also in absence of fungal cells, possibly due to their higher ability to produce extracellular matrix.

Apparent discrepancies between results from the biofilm biomass evaluation (Table 2) and the corresponding SEM images (e.g., Figures 8C,D differ significantly despite similar reduction rates) are due to differences in the corresponding controls. Despite SEM present a similar surface coverage of the controls, absorbance data indicate different biofilm biomass of the controls (Figure 2), most probably due to different biofilm thickness, not properly appreciable at SEM. Confocal laser scanning microscopy analysis carried out in a previous work demonstrated that the silicone coating with AC7BS expressed a detectable capacity in controlling *Candida* biofilm thickness after 48 h incubation (Ceresa et al., 2018).

No clear effect on the production of extracellular matrix by *Candida* was noted following the silicone precoating with R89BS or SL18. Indeed, the comparative analysis of *Candida* biofilm on the pre-coated discs with the controls did not show major differences in the amount of extracellular matrix. However, it has to be considered that SEM imaging in high vacuum brought to significant collapse of the matrix volume, limiting the sensitivity of this technique in revealing small changes in the matrix.

The strong association of *S. aureus* and *C. albicans* was indeed found in both untreated controls and pre-coated surfaces demonstrating the ability of *Candida* to provide an anchoring support for *S. aureus*. The anti-biofilm effect of the tested BSs could be therefore mainly related to the anti-adhesive properties rather than to a change in the microbial phenotypes including a reduction of the extracellular matrix production as reported by Gupta et al. (2019) using cholic acid-peptide conjugates.

Furthermore, the use of R89BS and SL18 as coating agents did not result in a clear modification of the *C. albicans* hyphal morphology. However, it would be interesting to evaluate in future whether these BSs, both in their soluble and coated form, are able to cause a delay on *Candida* yeast-to hyphal transition at the initial phases of biofilm formation, thus as reported for sophorolipids from *Starmerella bombicola* MTCC1910 (Haque et al., 2016) and a *Lactobacillus rhamnosus* (Tan et al., 2017).

Another important aspect is the biocompatibility of the AC7BS-, R89BS-, and SL18-coated silicone discs. It has been previously demonstrated that at concentrations lower than or equal to 0.2 mg/ml for R89BS and 0.5 mg/ml for AC7BS were not cytotoxic for MRC5 cells monolayers (Ceresa et al., 2018, 2019b). In this work, additional evaluations revealed no or negligible cytotoxicity on HaCaT cells when exposed to the BSs-coated discs eluates for up to 72 h, paving the way for further investigation toward future *in vivo* applications.

## Conclusion

In the present work, the activities of different BSs against yeast and bacterial biofilms were demonstrated through using a polymicrobial biofilm model able to support the growth of bacterial–fungal biofilms. The tests were conducted both under co-incubation and pre-coating conditions to evaluate, on the one hand, the inhibitory effect of BSs in solution against the polymicrobial biofilms and, on the other hand, the effectiveness of BSs as coating agents of medical devices to limit microbial infection. BSs successfully limited the formation of polymicrobial biofilms up to 3 days under both experimental conditions.

The obtained results, together with the non-toxic nature of BSs at the tested concentrations and the biocompatibility of BSs-coated discs, further support the idea of a possible applicability of these natural molecules in the biomedical field. In particular, BSs coating might be a promising strategy, supporting preventative infection measures and antimicrobial therapy, to reduce implant colonization and mitigate infections, thus prolonging the lifetime of implantable medical devices.

## Data Availability Statement

The raw data supporting the conclusions of this article will be made available by the authors, without undue reservation, to any qualified researcher.

## Author Contributions

CC and LF conceptualized the study. CC, FT, DM, and LF designed the experiments. PC, IB, and MD supervised the study. CC, FT, DM, EF, and ET performed the experiments. CC and MR collected data. MR carried out data analysis and statistics. PC, IB, MD, and LF contributed materials and analysis tools. CC, FT, MR, and LF wrote the original draft. CC, MR, FT, DM, IB, MD, and LF wrote, revised, and edited the manuscript. All authors read and approved the final manuscript.

## Conflict of Interest

The authors declare that the research was conducted in the absence of any commercial or financial relationships that could be construed as a potential conflict of interest.

## References

[B1] Adam B. Baillie G. S. Douglas L. J. (2002). Mixed species biofilms of *Candida albicans* and *Staphylococcus epidermidis*. *J. Med. Microbiol*. 51 344–349. 10.1099/0022-1317-51-4-344 11926741

[B2] Aleksic I. Petkovic M. Jovanovic M. Milivojevic D. Vasiljevic B. Nikodinovic-Runic J. (2017). Anti-biofilm properties of bacterial di-rhamnolipids and their semi-synthetic amide derivatives. *Front. Microbiol*. 8:2454. 10.3389/fmicb.2017.02454 29276509 PMC5727045

[B3] Arciola C. R. An Y. H. Campoccia D. Donati M. E. Montanaro L. (2005). Etiology of implant orthopedic infections: a survey on 1027 clinical isolates. *Int. J. Artif. Organs* 28 1091–1100. 10.1177/039139880502801106 16353115

[B4] Arciola C. R. Baldassarri L. Montanaro L. (2001). Presence of icaA and icaD genes and slime production in a collection of staphylococcal strains from catheter-associated infections. *J. Clin. Microbiol*. 39 2151–2156. 10.1128/JCM.39.6.2151-2156.2001 11376050 PMC88104

[B5] Banat I. M. Díaz De Rienzo M. A. Quinn G. A. (2014). Microbial biofilms: biosurfactants as antibiofilm agents. *Appl. Microbiol. Biotechnol*. 98 9915–9929. 10.1007/s00253-014-6169-6 25359476

[B6] Banat I. M. Franzetti A. Gandolfi I. Bestetti G. Martinotti M. G. Fracchia L. (2010). Microbial biosurfactants production, applications and future potential. *Appl. Microbiol. Biotechnol*. 87 427–444. 10.1007/s00253-010-2589-0 20424836

[B7] Brogden K. A. Guthmiller J. M. Taylor C. E. (2005). Human polymicrobial infections. *Lancet* 365 253–255. 10.1016/S0140-6736(05)70155-0 15652608 PMC7119324

[B8] Buch P. J. Chai Y. Goluch E. D. (2019). Treating polymicrobial infections in chronic diabetic wounds. *Clin. Microbiol. Rev*. 32:e00091-18. 10.1128/CMR.00091-18 30651226 PMC6431129

[B9] Budzynska A. Rozalska S. Sadowska B. Rozalska B. (2017). *Candida albicans/Staphylococcus aureus* dual-species biofilm as a target for the combination of essential oils and fluconazole or mupirocin. *Mycopathologia* 182 989–995. 10.1007/s11046-017-0192-y 28823093 PMC5684249

[B10] Burmølle M. Ren D. Bjarnsholt T. Sørensen S. J. (2014). Interactions in multispecies biofilms: do they actually matter? *Trends Microbiol.* 22 84–91. 10.1016/j.tim.2013.12.004 24440178

[B11] Carolus H. Van Dyck K. Van Dijck P. (2019). *Candida albicans* and *Staphylococcus* species: a threatening twosome. *Front. Microbiol.* 10:2162. 10.3389/fmicb.2019.02162 31620113 PMC6759544

[B12] Cassat J. E. Lee C. Y. Smeltzer M. S. (2007). Investigation of biofilm formation in clinical isolates of *Staphylococcus aureus*. *Methods Mol. Biol*. 391 127–144. 10.1007/978-1-59745-468-1_10 18025674 PMC4098860

[B13] Ceresa C. Fracchia L. Marchetti A. Rinaldi M. Bosetti M. (2019a). Injectable scaffolds enriched with silver to inhibit bacterial invasion in tissue regeneration. *Materials* (Basel) 12:E1931. 10.3390/ma12121931 31208032 PMC6631215

[B14] Ceresa C. Fracchia L. Williams M. Banat I. M. Díaz De Rienzo M. A. (2020). The effect of sophorolipids against microbial biofilms on medical-grade silicone. *J. Biotechnol*. 309 34–43. 10.1016/j.jbiotec.2019.12.019 31887325

[B15] Ceresa C. Rinaldi M. Chiono V. Carmagnola I. Allegrone G. Fracchia L. (2016). Lipopeptides from *Bacillus subtilis* AC7 inhibit adhesion and biofilm formation of *Candida albicans* on silicone. *Antonie Van Leeuwenhoek* 109 1375–1388. 10.1007/s10482-016-0736-z 27444239

[B16] Ceresa C. Rinaldi M. Fracchia L. (2017). Synergistic activity of antifungal drugs and lipopeptide AC7 against *Candida albicans* biofilm on silicone. *AIMS Bioeng*. 4 318–334. 10.3934/bioeng.2017.2.318

[B17] Ceresa C. Tessarolo F. Caola I. Nollo G. Cavallo M. Rinaldi M. (2015). Inhibition of *Candida albicans* adhesion on medical-grade silicone by a *Lactobacillus*-derived biosurfactant. *J. Appl. Microbiol.* 118 1116–1125. 10.1111/jam.12760 25644534

[B18] Ceresa C. Tessarolo F. Maniglio D. Caola I. Nollo G. Rinaldi M. (2018). Inhibition of *Candida albicans* biofilm by lipopeptide AC7 coated medical-grade silicone in combination with farnesol. *AIMS Bioeng*. 5 192–208. 10.3934/bioeng.2018.3.192

[B19] Ceresa C. Tessarolo F. Maniglio D. Tambone E. Carmagnola I. Fedeli E. (2019b). Medical-grade silicone coated with rhamnolipid R89 is effective against *Staphylococcus* spp. *Biofilms Mol.* 24:E3843. 10.3390/molecules24213843 31731408 PMC6864460

[B20] Chen L. Wen Y. M. (2011). The role of bacterial biofilm in persistent infections and control strategies. *Int. J. Oral. Sci*. 3 66–73. 10.4248/IJOS11022 21485310 PMC3469879

[B21] Clinton A. Carter T. (2015). Chronic wound biofilms: pathogenesis and potential therapies. *Lab Med*. 46 277–284. 10.1309/LMBNSWKUI4JPN7SO 26489671

[B22] Comoglio F. Fracchia L. Rinaldi M. (2013). Bayesian inference from count data using discrete uniform priors. *PLoS One* 8:e74388. 10.1371/journal.pone.0074388 24116003 PMC3792115

[B23] Cuesta A. I. Jewtuchowicz V. Brusca M. I. Nastri M. L. Rosa A. C. (2010). Prevalence of *Staphylococcus* spp and *Candida* spp in the oral cavity and periodontal pockets of periodontal disease patients. *Acta Odontol. Latinoam*. 23 20–26. 20645638

[B24] de Alteriis E. Lombardi L. Falanga A. Napolano M. Galdiero S. Siciliano A. (2018). Polymicrobial antibiofilm activity of the membranotropic peptide gH625 and its analogue. *Microb. Pathog*. 125 189–195. 10.1016/j.micpath.2018.09.027 30227230

[B25] Diaz De Rienzo M. A. Dolman B. Guzman F. Kaisermann C. Winterburn J. Banat I. M. (2014). Antimicrobial properties of sophorolipids produced by *Candida bombicola* ATCC 22214 against gram positive and Gram-negative bacteria. *New Biotechnol.* 31 S66–S67. 10.1016/j.nbt.2014.05.1764

[B26] Diaz De Rienzo M. A. Stevenson P. S. Marchant R. Banat I. M. (2016). Effect of biosurfactants on *Pseudomonas aeruginosa* and *Staphylococcus aureus* biofilms in a BioFlux channel. *Appl. Microbiol. Biotechnol.* 100 5773–5779. 10.1007/s00253-016-7310-5 26825819 PMC4909806

[B27] Elshikh M. Marchant R. Banat I. M. (2016). Biosurfactants: promising bioactive molecules for oral-related health applications. *FEMS Microbiol. Lett.* 363:fnw213. 10.1093/femsle/fnw213 27619892

[B28] Elshikh M. Moya-Ramírez I. Moens H. Roelants S. Soetaert W. Marchant R. (2017). Rhamnolipids and lactonic sophorolipids: natural antimicrobial surfactants for oral hygiene. *J. Appl. Microbiol.* 123 1111–1123. 10.1111/jam.13550 28766815

[B29] Fracchia L. Banat J. J. Cavallo M. Ceresa C. Banat I. M. (2015). Potential therapeutic applications of microbial surface-active compounds. *AIMS Bioeng*. 2 144–162. 10.3934/bioeng.2015.3.144

[B30] Fracchia L. Ceresa C. Banat I. M. (2019). “Biosurfactants in cosmetic, biomedical and pharmaceutical industry,” in *Microbial Biosurfactants and their Environmental and Industrial Applications*, eds Banat I. M. Thavasi R. (Boca Raton, FL: CRS Press), 258–288. 10.1201/b21950-11

[B31] Francolini I. Donelli G. (2010). Prevention and control of biofilm-based medical-device-related infections. *FEMS Immunol. Med. Microbiol.* 59 227–238. 10.1111/j.1574-695X.2010.00665.x 20412300

[B32] Francolini I. Vuotto C. Piozzi A. Donelli G. (2017). Antifouling and antimicrobial biomaterials: an overview. *APMIS* 125 392–417. 10.1111/apm.12675 28407425

[B33] Ghensi P. Bettio E. Maniglio D. Bonomi E. Piccoli F. Gross S. (2019). Dental implants with anti-biofilm properties: a pilot study for developing a new sericin-based coating. *Materials* (Basel). 12:E2429. 10.3390/ma12152429 31366076 PMC6695694

[B34] Gupta N. Haque A. Mukhopadhyay G. Narayan R. P. Prasad R. (2005). Interactions between bacteria and *Candida* in the burn wound. *Burns* 31 375–378. 10.1016/j.burns.2004.11.012 15774298

[B35] Gupta S. Raghuwanshi N. Varshney R. Banat I. M. Srivastava A. K. Pruthi P. A. (2017). Accelerated in vivo wound healing evaluation of microbial glycolipid containing ointment as a transdermal substitute. *Biomed. Pharmacother*. 94 1186–1196. 10.1016/j.biopha.2017.08.010 28830069

[B36] Gupta S. Thakur J. Pal S. Gupta R. Mishra D. Kumar S. (2019). Cholic acid-peptide conjugates as potent antimicrobials against interkingdom polymicrobial biofilms. *Antimicrob Agents Chemother*. 63:e00520-19. 10.1128/AAC.00520-19 31427303 PMC6811435

[B37] Haque F. Alfatah M. Ganesan K. Bhattacharyya M. S. (2016). Inhibitory effect of sophorolipid on *Candida albicans* biofilm formation and hyphal growth. *Sci. Rep.* 6:23575. 10.1038/srep23575 27030404 PMC4876995

[B38] Harriott M. M. Noverr M. C. (2009). *Candida albicans* and *Staphylococcus aureus* form polymicrobial biofilms: effects on antimicrobial resistance. *Antimicrob. Agents Chemother*. 53 3914–3922. 10.1128/AAC.00657-09 19564370 PMC2737866

[B39] Harriott M. M. Noverr M. C. (2011). Importance of *Candida*-bacterial polymicrobial biofilms in disease. *Trends Microbiol*. 19 557–563. 10.1016/j.tim.2011.07.004 21855346 PMC3205277

[B40] Hrubanova K. Krzyzanek V. Nebesarova J. Ruzicka F. Pilat Z. Samek O. (2018). Monitoring *Candida parapsilosis* and *Staphylococcus epidermidis* biofilms by a combination of scanning electron microscopy and raman spectroscopy. *Sensors (Basel)* 18:E4089. 10.3390/s18124089 30469521 PMC6308600

[B41] Irorere U. V. Kwiencien M. Tripathi L. Cobice D. McClean S. Marchant R. (2019). Quorum sensing as a potential target for increased production of rhamnolipid biosurfactant in *Burkholderia thailandensis* E264. *Appl. Microbiol. Biotechnol*. 103 6505–6517. 10.1007/s00253-019-09942-5 31222386 PMC6667413

[B42] Janek T. Krasowska A. Czyżnikowska Ż Łukaszewicz M. (2018). Trehalose lipid biosurfactant reduces adhesion of microbial pathogens to polystyrene and silicone surfaces: an experimental and computational approach. *Front. Microbiol*. 9:2441. 10.3389/fmicb.2018.02441 30386313 PMC6198247

[B43] John T. Rajpurkar A. Smith G. Fairfax M. Triest J. (2007). Antibiotic pretreatment of hydrogel ureteral stent. *J. Endourol.* 21 1211–1216. 10.1089/end.2007.9904 17949328

[B44] Juma A. Lemoine P. Simpson A. B. J. Murray J. O’Hagan B. M. G. Naughton P. J. (2020). Microscopic investigation of the combined use of antibiotics and biosurfactants on methicillin resistant *Staphylococcus aureus*. *Front. Microbiol*. 11:1477. 10.3389/fmicb.2020.01477 32733412 PMC7358407

[B45] Khatoon Z. McTiernan C. D. Suuronen E. J. Mah T. F. Alarcon E. I. (2018). Bacterial biofilm formation on implantable devices and approaches to its treatment and prevention. *Heliyon* 4:e01067. 10.1016/j.heliyon.2018.e01067 30619958 PMC6312881

[B46] Koo H. Allan R. N. Howlin R. P. Stoodley P. Hall-Stoodley L. (2017). Targeting microbial biofilms: current and prospective therapeutic strategies. *Nat. Rev. Microbiol.* 15 740–755. 10.1038/nrmicro.2017.99 28944770 PMC5685531

[B47] Liu H. Chen H. Sun Y. Zhang X. Lu H. Li J. (2019). Characterization of the mechanism and impact of staphylokinase on the formation of *Candida albicans* and *Staphylococcus aureus* polymicrobial biofilms. *J. Med. Microbiol.* 68 355–367. 10.1099/jmm.0.000914 30628885

[B48] Lydon H. L. Baccile N. Callaghan B. Marchant R. Mitchell C. A. Banat I. M. (2017). Adjuvant antibiotic activity of acidic sophorolipids with potential for facilitating wound healing. *Antimicrob. Agents Chemother.* 61:e02547-16. 10.1128/AAC.02547-16 28242666 PMC5404594

[B49] Marculescu C. E. Cantey J. R. (2008). Polymicrobial prosthetic joint infections: risk factors and outcome. *Clin. Orthop. Relat. Res*. 466 1397–1404. 10.1007/s11999-008-0230-7 18421538 PMC2384015

[B50] Mihai M. M. Holban A. M. Giurcaneanu C. Popa L. G. Oanea R. M. Lazar V. (2015). Microbial biofilms: impact on the pathogenesis of periodontitis, cystic fibrosis, chronic wounds and medical device-related infections. *Curr. Top. Med. Chem.* 15 1552–1576. 10.2174/1568026615666150414123800 25877092

[B51] Naughton P. Marchant R. Naughton V. Banat I. (2019). Microbial biosurfactants: current trends and applications in agricultural and biomedical industries. *J. Appl. Microbiol*. 127 12–28. 10.1111/jam.14243 30828919

[B52] Omar A. Wright J. B. Schultz G. Burrell R. Nadworny P. (2017). Microbial biofilms and chronic wounds. *Microorganisms* 5:E9. 10.3390/microorganisms5010009 28272369 PMC5374386

[B53] Pammi M. Liang R. Hicks J. Mistretta T. A. Versalovic J. (2013). Biofilm extracellular DNA enhances mixed species biofilms of *Staphylococcus epidermidis* and *Candida albicans*. *BMC Microbiol*. 13:257. 10.1186/1471-2180-13-257 24228850 PMC3833181

[B54] Papa R. Selan L. Parrilli E. Tilotta M. Sannino F. Feller G. (2015). Activities from marine cold adapted bacteria against *Staphylococci* and *Pseudomonas aeruginosa*. *Front. Microbiol*. 6:1333. 10.3389/fmicb.2015.01333 26696962 PMC4677098

[B55] Paraszkiewicz K. Moryl M. Płaza G. Bhagat D. Satpute S. K. Bernat P. (2019). Surfactants of microbial origin as antibiofilm agents. *Int. J. Environ. Health Res*. 11 1–20. 10.1080/09603123.2019.1664729 31509014

[B56] Percival S. L. Suleman L. Vuotto C. Donelli G. (2015). Healthcare-associated infections, medical devices and biofilms: risk, tolerance and control. *J. Med. Microbiol.* 64 323–334. 10.1099/jmm.0.000032 25670813

[B57] Pompilio A. Di Bonaventura G. (2018). Microbial biofilm: a “sticky” problem. *Microbiol. Med.* 33:7851 10.4081/mm.2018.7851

[B58] Qu Y. Locock K. Verma-Gaur J. Hay I. D. Meagher L. Traven A. (2016). Searching for new strategies against polymicrobial biofilm infections: guanylated polymethacrylates kill mixed fungal/bacterial biofilms. *J. Antimicrob. Chemother*. 71 413–421. 10.1093/jac/dkv334 26490013

[B59] Quinn G. A. Maloy A. P. Banat M. M. Banat I. M. (2013). A comparison of effects of broad-spectrum antibiotics and biosurfactants on established bacterial biofilms. *Curr. Microbiol*. 67 614–623. 10.1007/s00284-013-0412-8 23783562

[B60] Ramasamy M. Lee J. (2016). Recent nanotechnology approaches for prevention and treatment of biofilm-associated infections on medical devices. *Biomed. Res. Int.* 2016:1851242. 10.1155/2016/1851242 27872845 PMC5107826

[B61] R Core Team (2019). *R: A Language and Environment for Statistical Computing.* Vienna: R Foundation for Statistical Computing Available online at: https://www.Rproject.org/

[B62] Rodrigues L. R. Banat I. M. van der Mei H. C. Teixeira J. A. Oliveira R. (2006). Interference in adhesion of bacteria and yeasts isolated from explanted voice prostheses to silicone rubber by rhamnolipid biosurfactants. *J. Appl. Microbiol*. 100 470–480. 10.1111/j.1365-2672.2005.02826.x 16478486

[B63] Rodrigues L. R. Teixeira J. A. (2010). Biomedical and therapeutic applications of biosurfactants. *Adv. Exp. Med. Biol*. 672 75–87. 10.1007/978-1-4419-5979-9_6 20545275

[B64] Rodrigues M. E. Gomes F. Rodrigues C. F. (2019). *Candida* spp./bacteria mixed biofilms. *J. Fungi* (Basel). 6:5. 10.3390/jof6010005 31861858 PMC7151131

[B65] Rodríguez-López L. López-Prieto A. Lopez-Álvarez M. Pérez-Davila S. Serra J. González P. (2020). Characterization and cytotoxic effect of biosurfactants obtained from different sources. *ACS Omega* 48 31381–31390. 10.1021/acsomega.0c04933 33324849 PMC7726928

[B66] Rosenberg M. Gutnick D. Rosenberg E. (1980). Adherence of bacteria to hydrocarbons: a simple method for measuring cell-surface hydrophobicity. *FEMS Microbiol. Lett.* 9 29–33. 10.1111/j.1574-6968.1980.tb05599.x

[B67] Sardi J. C. Scorzoni L. Bernardi T. Fusco-Almeida A. M. Mendes Giannini M. J. (2013). *Candida* species: current epidemiology, pathogenicity, biofilm formation, natural antifungal products and new therapeutic options. *J. Med. Microbiol*. 62 10–24. 10.1099/jmm.0.045054-0 23180477

[B68] Satpute S. K. Banpurkar A. G. Banat I. M. Sangshetti J. N. Patil R. H. Gade W. N. (2016). Multiple roles of biosurfactants in biofilms. *Curr. Pharm. Des*. 22 1429–1448. 10.2174/1381612822666160120152704 26786675

[B69] Satpute S. K. Mone N. S. Das P. Banat I. M. Banpurkar A. G. (2019). Inhibition of pathogenic bacterial biofilms on PDMS based implants by *L. acidophilus* derived biosurfactant. *BMC Microbiol*. 19:39. 10.1186/s12866-019-1412-z 30760203 PMC6374892

[B70] Satpute S. K. Mone N. S. Das P. Banpurkar A. G. Banat I. M. (2018). *Lactobacillus acidophilus* derived biosurfactant as a biofilm inhibitor: a promising investigation using microfluidic approach. *Appl. Sci*. 8:1555 10.3390/app8091555

[B71] Scaffaro R. Lopresti F. D’Arrigo M. Marino A. Nostro A. (2018). Efficacy of poly(lactic acid)/carvacrol electrospun membranes against *Staphylococcus aureus* and *Candida albicans* in single and mixed cultures. *Appl. Microbiol. Biotechnol*. 102 4171–4181. 10.1007/s00253-018-8879-7 29536146

[B72] Sharma D. Misba L. Khan A. U. (2019). Antibiotics versus biofilm: an emerging battleground in microbial communities. *Antimicrob. Resist. Infect. Control* 8:76. 10.1186/s13756-019-0533-3 31131107 PMC6524306

[B73] Sharma D. Saharan B. S. (2016). Functional characterization of biomedical potential of biosurfactant produced by *Lactobacillus helveticus*. *Biotechnol. Rep. (Amst.)* 11 27–35. 10.1016/j.btre.2016.05.001 28352537 PMC5042301

[B74] Signoretto C. Marchi A. Bertoncelli A. Burlacchini G. Milli A. Tessarolo F. (2013). Effects of mushroom and chicory extracts on the shape, physiology and proteome of the cariogenic bacterium *Streptococcus mutans*. *BMC Complement. Altern. Med*. 13:117. 10.1186/1472-6882-13-117 23714053 PMC3672068

[B75] Simms A. A. Naughton P. J. Marchant R. Banat I. M. (2020). Microbial biosurfactants in cosmetic and personal skincare pharmaceutical formulations. *Pharmaceutics* 12:1099 10.3390/pharmaceutics12111099 PMC7696787 33207832

[B76] Song R. Murphy M. Li C. Ting K. Soo C. Zheng Z. (2018). Current development of biodegradable polymeric materials for biomedical applications. *Drug Des. Devel. Ther.* 12 3117–3145. 10.2147/DDDT.S165440 30288019 PMC6161720

[B77] Tan Y. Leonhard M. Moser D. Ma S. Schneider-Stickler B. (2019). Antibiofilm efficacy of curcumin in combination with 2-aminobenzimidazole against single- and mixed-species biofilms of *Candida albicans* and *Staphylococcus aureus*. *Colloids Surf. B Biointerfaces* 174 28–34. 10.1016/j.colsurfb.2018.10.079 30412864

[B78] Tan Y. Leonhard M. Moser D. Schneider-Stickler B. (2017). Inhibition activity of *Lactobacilli* supernatant against fungal-bacterial multispecies biofilms on silicone. *Microb. Pathog*. 113 197–201. 10.1016/j.micpath.2017.10.051 29111321

[B79] Teo A. J. T. Mishra A. Park I. Kim Y.-J. Park W.-T. Yoon Y.-J. (2016). Polymeric biomaterials for medical implants and devices. *ACS Biomater. Sci. Eng.* 2 454–472. 10.1021/acsbiomaterials.5b00429 33465850

[B80] Tessarolo F. Caola I. Fedel M. Stacchiotti A. Caciagli P. Guarrera G. M. (2007). Different experimental protocols for decontamination affect the cleaning of medical devices. A preliminary electron microscopy analysis. *J. Hosp. Infect*. 65 326–333. 10.1016/j.jhin.2006.10.015 17241696

[B81] Tsui C. Kong E. F. Jabra-Rizk M. A. (2016). Pathogenesis of *Candida albicans* biofilm. *Pathog. Dis.* 74:ftw018. 10.1093/femspd/ftw018 26960943 PMC5975230

[B82] Valenza G. Tappe D. Turnwald D. Frosch M. Konig C. Hebestreit H. (2008). Prevalence and antimicrobial susceptibility of microorganisms isolated from sputa of patients with cystic fibrosis. *J. Cyst. Fibros*. 7 123–127. 10.1016/j.jcf.2007.06.006 17693140

[B83] Vasilev K. Griesser S. S. Griesser H. J. (2011). Antibacterial surfaces and coatings produced by plasma techniques. *Plasma Process. Polym*. 8 1010–1023. 10.1002/ppap.201100097

[B84] Villa F. Cappitelli F. (2013). Plant-derived bioactive compounds at sub-lethal concentrations: towards smart biocide-free antibiofilm strategies. *Phytochem. Rev*. 12 245–254. 10.1007/s11101-013-9286-4

[B85] von Eiff C. Arciola C. R. Montanaro L. Becker K. Campoccia D. (2006). Emerging *Staphylococcus* species as new pathogens in implant infections. *Int. J. Artif. Organs* 29 360–367. 10.1177/039139880602900405 16705604

[B86] Wang M. Tang T. (2018). Surface treatment strategies to combat implant-related infection from the beginning. *J. Orthop. Translat*. 17 42–54. 10.1016/j.jot.2018.09.001 31194031 PMC6551355

[B87] Wang Y. Jayan G. Patwardhan D. Phillips K. S. (2017). “Antimicrobial and anti-biofilm medical devices: public health and regulatory science challenges,” in *Antimicrobial Coatings and Modifications on Medical Devices*, eds Zhang Z. Wagner V. (Cham: Springer), 37–75. 10.1007/978-3-319-57494-3_2

[B88] Weiland-Bräuer N. Malek I. Schmitz R. A. (2019). Metagenomic quorum quenching enzymes affect biofilm formation of *Candida albicans* and *Staphylococcus epidermidis*. *PLoS One* 14:e0211366. 10.1371/journal.pone.0211366 30689669 PMC6349329

[B89] Williams D. L. Bloebaum R. D. (2010). Observing the biofilm matrix of *Staphylococcus epidermidis* ATCC 35984 grown using the CDC biofilm reactor. *Microsc. Microanal*. 16 143–152. 10.1017/S143192760999136X 20205969

[B90] Xian W. (2009). “Module III: biocompatibility testing: cytotoxicity and adhesion,” in *A Laboratory Course in Biomaterials*, ed. Taylor & Francis Group (Boca Raton, FL: CRC Press), 99–128. 10.1201/b15832

[B91] Zilberman M. Elsner J. J. (2008). Antibiotic-eluting medical devices for various applications. *J. Control Release* 130 202–215. 10.1016/j.jconrel.2008.05.020 18687500

