# Supplementary figures and images for: Inhibitory Effects of Lipopeptides and Glycolipids on C. albicans–Staphylococcus spp. Dual-Species Biofilms
Source: Front Microbiol. 2021 Jan 13;11:545654. doi: 10.3389/fmicb.2020.545654 (PMC7838448; doi:10.3389/fmicb.2020.545654)

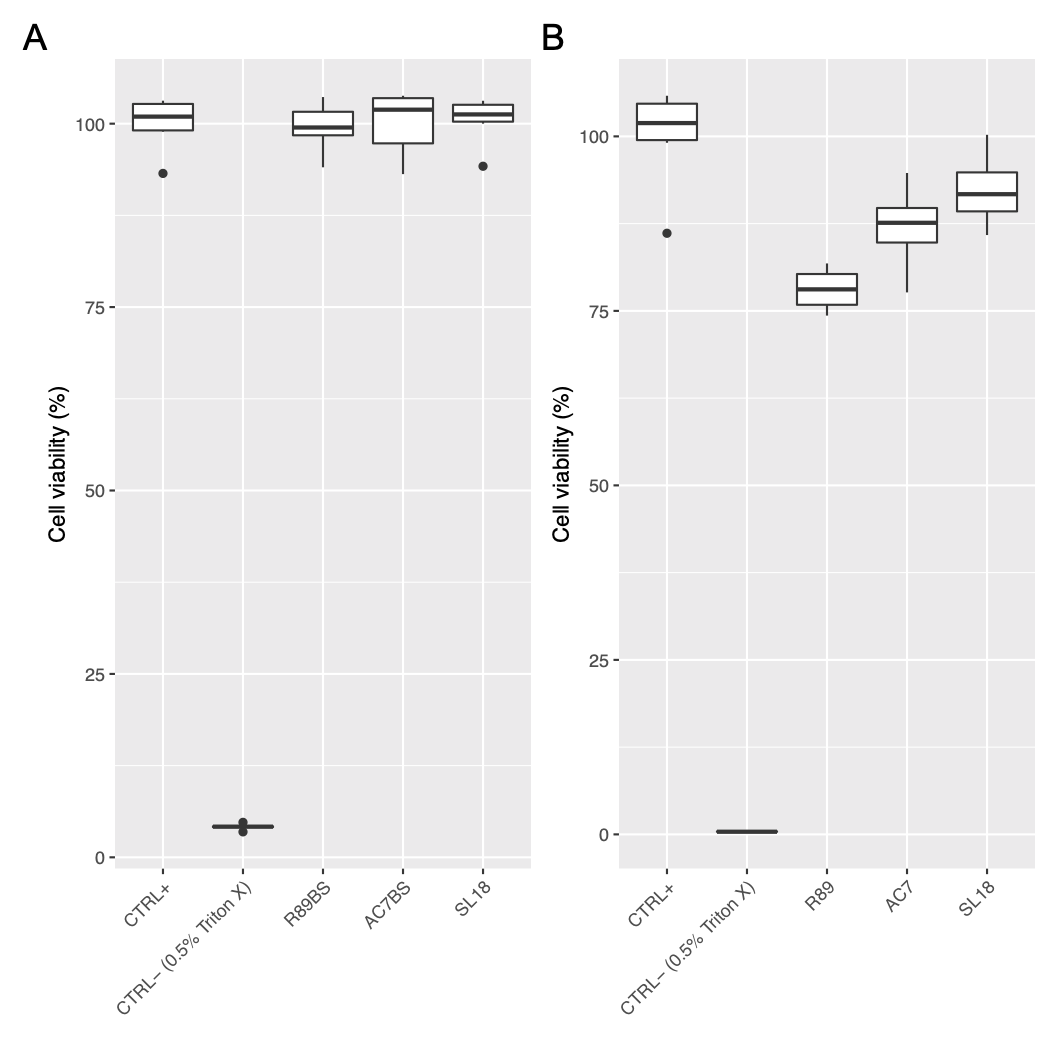

Supplement: Supplementary file 3 [file Image_3.TIFF]
