# Supplementary material for: Inhibitory Effects of Lipopeptides and Glycolipids on C. albicans–Staphylococcus spp. Dual-Species Biofilms
Source: Front Microbiol. 2021 Jan 13;11:545654. doi: 10.3389/fmicb.2020.545654 (PMC7838448; doi:10.3389/fmicb.2020.545654)
Supplement: Supplementary file 4 [file Table_1.DOCX]

Supplementary Material

| Time (h) | Strain | Treatment | | | |
| --- | --- | --- | --- | --- | --- |
|  |  | CTRL | AC7BS | R89BS | SL18 |
| 24 | *C. albicans* | 3.42 × 10^5^ | 1.39 × 10^4^ | 1.07 × 10^4^ | 1.18 × 10^4^ |
|  | *S. aureus* | 2.48 × 10^7^ | 2.93 × 10^5^ | 2.08 × 10^5^ | 2.78 × 10^5^ |
| 48 | *C. albicans* | 7.25 × 10^5^ | 2.91 × 10^4^ | 1.01 × 10^4^ | 1.61 × 10^4^ |
|  | *S. aureus* | 5.03 × 10^7^ | 1.80 × 10^6^ | 5.70 × 10^5^ | 1.71 × 10^6^ |
| 72 | *C. albicans* | 1.46 × 10^6^ | 2.46 × 10^5^ | 1.04 ×10^4^ | 2.65 × 10^4^ |
|  | *S. aureus* | 1.09 × 10^8^ | 1.68 × 10^7^ | 3.37 × 10^6^ | 5.58 × 10^6^ |

**Supplementary Table 1. Mean *C. albicans* and *S. aureus* concentrations, expressed as CFU/disc, in *C. albicans* - *S. aureus* dual-species biofilms on the surface of silicone discs.**

| Time (h) | Strain | Treatment | | |
| --- | --- | --- | --- | --- |
|  |  | CTRL | R89BS | SL18 |
| 24 | *C. albicans* | 4.33 × 10^5^ | 3.08 × 10^4^ | 3.17 × 10^4^ |
|  | *S. epidermidis* | 2.14 × 10^8^ | 3.03 × 10^6^ | 5.44 × 10^6^ |
| 48 | *C. albicans* | 8.00 × 10^5^ | 4.86 × 10^4^ | 4.35 × 10^4^ |
|  | *S. epidermidis* | 5.03 × 10^8^ | 2.68 × 10^7^ | 6.33 × 10^7^ |
| 72 | *C. albicans* | 1.50 × 10^6^ | 1.58 × 10^5^ | 3.83 × 10^5^ |
|  | *S. epidermidis* | 9.42 × 10^8^ | 8.67 × 10^7^ | 2.18 × 10^8^ |

**Supplementary Table 2. Mean *C. albicans* and *S. epidermidis* concentrations, expressed as CFU/disc, in *C. albicans* - *S. epidermidis* dual-species biofilms on the surface of silicone discs.**

**Supplementary Figure 1.** **Scanning electron microscopy images of the dual-species biofilms formed on the silicone discs surface at 24h.** *C. albicans* - *S. aureus* on the left column, *C. albicans* - *S. epidermidis* on the right column. Different surface pre-coating treatments are presented: untreated controls (top row), rhamnolipid R89BS (middle raw) and sophorolipid SL18 (bottom row) treated discs. Original magnification: 4000x.

**Supplementary Figure 2: Scanning electron microscopy images of the dual-species biofilms formed on the silicone discs surface at 48h.** *C. albicans* - *S. aureus* on the left column, *C. albicans* - *S. epidermidis* on the right column. Different surface pre-coating treatments are presented: untreated controls (top row), rhamnolipid R89BS (middle raw) and sophorolipid SL18 (bottom row) treated discs. Original magnification: 4000x.

**Supplementary Figure 3: Cytotoxicity of BSs-coated SEDs on spontaneously immortalized human skin keratinocyte (HaCaT) at 24h and 72h.** Positive control (CTRL+) is represented by cells in standard growth medium and negative control (CTRL-) is represented by fully lysate cells (0.5% Triton X).
